# Humanized liver TK-NOG mice with functional deletion of hepatic murine cytochrome P450s as a model for studying human drug metabolism

**DOI:** 10.1038/s41598-022-19242-0

**Published:** 2022-09-01

**Authors:** Shotaro Uehara, Yuichi Iida, Miyuki Ida-Tanaka, Motohito Goto, Kenji Kawai, Masafumi Yamamoto, Yuichiro Higuchi, Satoshi Ito, Riichi Takahashi, Hidetaka Kamimura, Mamoru Ito, Hiroshi Yamazaki, Mitsuo Oshimura, Yasuhiro Kazuki, Hiroshi Suemizu

**Affiliations:** 1 Liver Engineering Laboratory, Department of Applied Research for Laboratory Animals, Central Institute for Experimental Animals (CIEA), 3-25-12 Tonomachi, Kawasaki-ku, Kawasaki, 210-0821 Japan; 2 Chromosome Engineering Research Center (CERC), Tottori University, Yonago, Japan; 3 Animal Resource and Technical Research Center, CIEA, Kawasaki, Japan; 4 Pathological Analysis Center, CIEA, Kawasaki, Japan; 5 ICLAS Monitoring Center, CIEA, Kawasaki, Japan; 6 Drug Development Solutions Center, Sekisui Medical Co., Ltd., Ibaraki, Japan; 7 Laboratory of Drug Metabolism and Pharmacokinetics, Showa Pharmaceutical University, Machida, Japan; 8 Department of Chromosome Biomedical Engineering, School of Life Science, Faculty of Medicine, Tottori University, Yonago, Japan; 9 Present Address: Department of Immunology, Shimane University Faculty of Medicine, Izumo, Japan; 10 Present Address: Laboratory Animal Research Department, CIEA, Kawasaki, Japan

**Keywords:** Preclinical research, Animal disease models

## Abstract

Chimeric TK-NOG mice with a humanized liver (normal Hu-liver) are a unique animal model for predicting drug metabolism in humans. However, residual mouse hepatocytes occasionally prevent the precise evaluation of human drug metabolism. Herein, we developed a novel humanized liver TK-NOG mouse with a conditional knockout of liver-specific cytochrome P450 oxidoreductase (POR cKO Hu-liver). Immunohistochemical analysis revealed only a few POR-expressing cells around the portal vein in POR cKO mouse livers. NADPH-cytochrome c reductase and cytochrome P450 (P450)-mediated drug oxidation activity in liver microsomes from POR cKO mice was negligible. After the intravenous administration of *S*-warfarin, high circulating and urinary levels of *S*-7-hydroxywarfarin (a major human metabolite) were observed in POR cKO Hu-liver mice. Notably, the circulating and urinary levels of *S*-4′-hydroxywarfarin (a major warfarin metabolite in mice) were much lower in POR cKO Hu-liver mice than in normal Hu-liver mice. POR cKO Hu-liver mice with minimal interference from mouse hepatic P450 oxidation activity are a valuable model for predicting human drug metabolism.

## Introduction

Identifying the major human metabolites of drug candidates is essential for drug development, and as such, metabolites are a common cause of drug hepatoxicity. Although preclinical safety assessment in experimental animals is essential for estimating the toxicity of drug candidates, interspecies differences in drug metabolism compromise the accuracy of these model systems. In vitro liver preparations from humans (e.g., hepatocytes, S9 fractions, and microsomes) are widely used for preliminary assessment but are often inadequate with regard to the prediction of drug metabolism with low-turnover or multi-step reactions^1^. Thus, a better preclinical model is required to obtain more accurate insights into the human metabolic profile early in the drug development process.

Chimeric mice with humanized livers are an attractive model for predicting drug metabolism in humans because the liver is the major drug-metabolizing organ. Chimeric TK-NOG mice with humanized livers were constructed through the transplantation of human hepatocytes into TK-NOG transgenic mice, which express herpes simplex virus type 1 thymidine kinase (HSVtk) in the liver under the regulation of a mouse albumin enhancer/promoter^2^. Human Phase I and II drug-metabolizing enzymes were expressed in the repopulated liver of humanized mice^2^. These mice have been successfully used to predict drug metabolite levels in humans. Previous studies included human-specific 3-hydroxylation and subsequent *O*-glucuronidation of desloratadine^3^, methyl-hydroxylation, and subsequent oxidation to produce carboxylic acid of tolbutamide by multiple drug-metabolizing enzymes^4^, as well as UDP-glucuronosyltransferase 1A4-mediated N2-glucuronidation^5^. However, even humanized liver mice with high hepatic replacement (> 90%) harbored residual mouse hepatocytes. These mouse hepatocytes potentially influence the drug metabolic profile in humanized liver mice because, in many cases, the intrinsic clearance within mouse liver microsomal fractions is higher than that in human liver microsomal fractions^6^.

Cytochrome P450s are major catalysts involved in drug metabolism and bioactivation, accounting for almost 70–80% of the total drug metabolism^7^. Variations in P450 function determine interspecies differences in drug metabolism and pharmacokinetics. In this regard, mice have more functional P450 genes (72 P450 genes) than humans (57 P450 genes)^8^. The catalytic function of mouse P450s is yet to be elucidated and compared with that of human P450s, which further complicates our understanding of interspecies differences in drug metabolism between humans and mice. P450 oxidoreductase (POR) is a diflavin enzyme responsible for electron donation to a large number of P450 enzymes^9^. POR-null mice exhibit early embryonic lethality at E10.5, as P450 enzymes are involved in various endogenous metabolic pathways for embryonic development^10^. Hepatic POR-null mice showed loss or low levels of hepatic P450 activity^11,12^. Therefore, liver-specific POR knockout mice may represent a novel platform for developing a better humanized liver model with minimal interference of mouse hepatic P450 activity. Barzi et al. reported the functional superiority in the conditional knockout of the NADPH-P450 oxidoreductase gene combined with FRG (*Fah*^−/−^/*Rag2*^−/−^/*Il2rg*^−/−^) humanized liver mice (PIRF mice)^13^. However, the PIRF mouse model is a slightly complicated system in which CRE recombinase is expressed by adenoviral transduction to conditionally delete the *Por* gene in the liver.

In this study, we developed a novel POR conditional knockout humanized liver TK-NOG mouse (POR cKO Hu-liver) with negligible endogenous hepatic P450 activity and a standard intrahepatic human P450 activity. We characterized the metabolic properties of POR cKO humanized liver mice based on species-dependent *S*-warfarin metabolism.

## Results

### Characterization of POR conditional knockout mice

**Figure 1:**
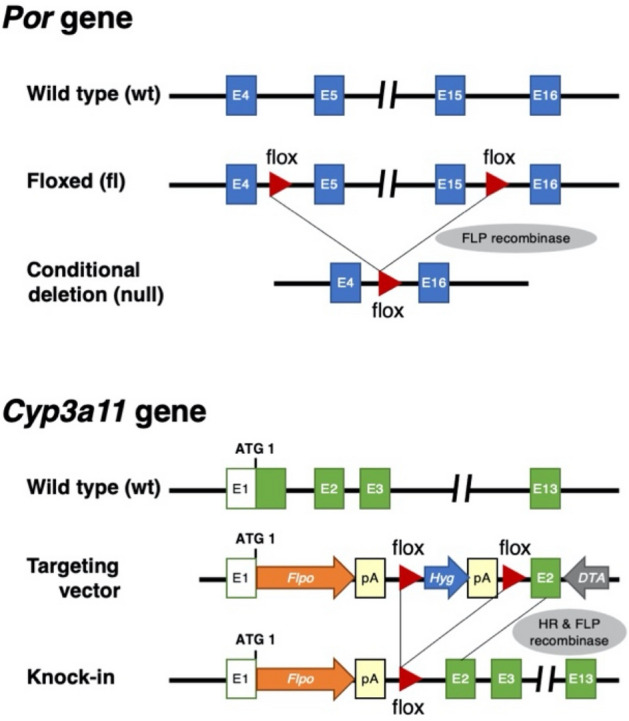
Schematic representation of deleted loci of *Por* gene and *Flpo* recombinase knocked-in loci of *Cyp3a11* in the POR conditional knockout mice. *pA* poly A signal, *HR* homologous recombination.

**Figure 2:**
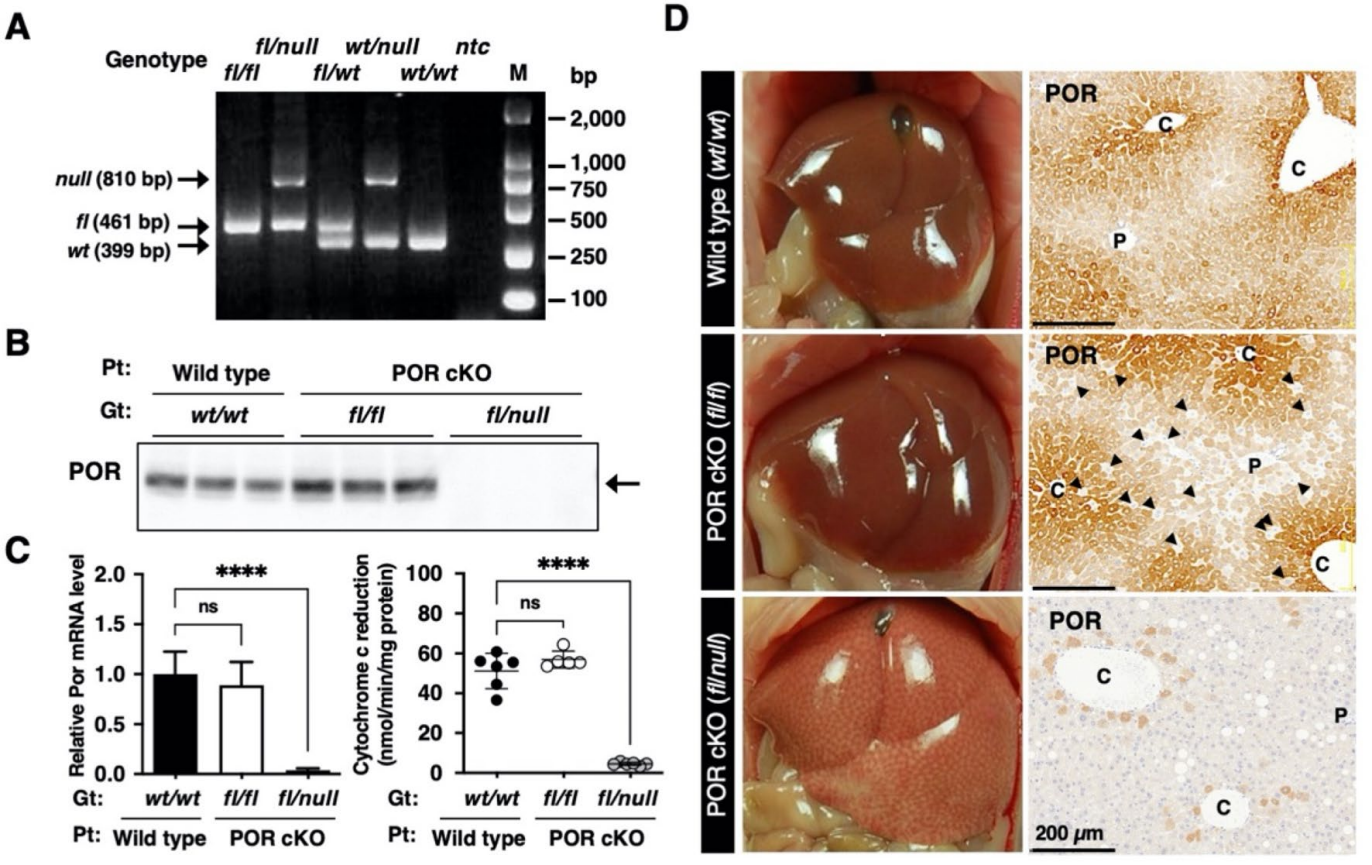
Generation of POR conditional knockout mice. (**A**) Representative PCR genotyping result for POR conditional knockout mouse. *fl*, floxed allele, *wt*, wild type, *ntc*, no template control. Full-length gel image was presented in Supplemental Fig. 6. (**B**) Western blot analysis of POR protein in liver microsomes from wildtype, POR cKO *fl/fl*, and POR cKO *fl/null* mice. Protein expression of mouse POR (arrow) assessed using immunoblotting with polyclonal antisera against POR. Gt, genotype, Pt, phenotype. Full-length blot image was presented in Supplemental Fig. 7. (**C**) Expression level of POR mRNA in the livers of wild-type mice (n = 6), POR cKO *fl/fl* (n = 9), and POR cKO *fl/null* mice (n = 6) were measured using qRT-PCR. The NADPH-cytochrome c reducing activity was determined to estimate POR activity in mouse liver microsomes. Data are presented as the mean ± SD. Statistically significant difference when compared with that in wild-type (TK-NOG) mice; ns, not significant, ****p < 0.0001. (**D**) Immunohistochemical staining of POR protein in livers from wild type, POR cKO *fl/fl*, and POR cKO *fl/null* mice. P, portal tract, C, central vein. Scale bar, 200 μm. Arrow-heads in POR cKO *fl/fl* panel indicate typical cells not express the POR protein.

**Figure 3:**
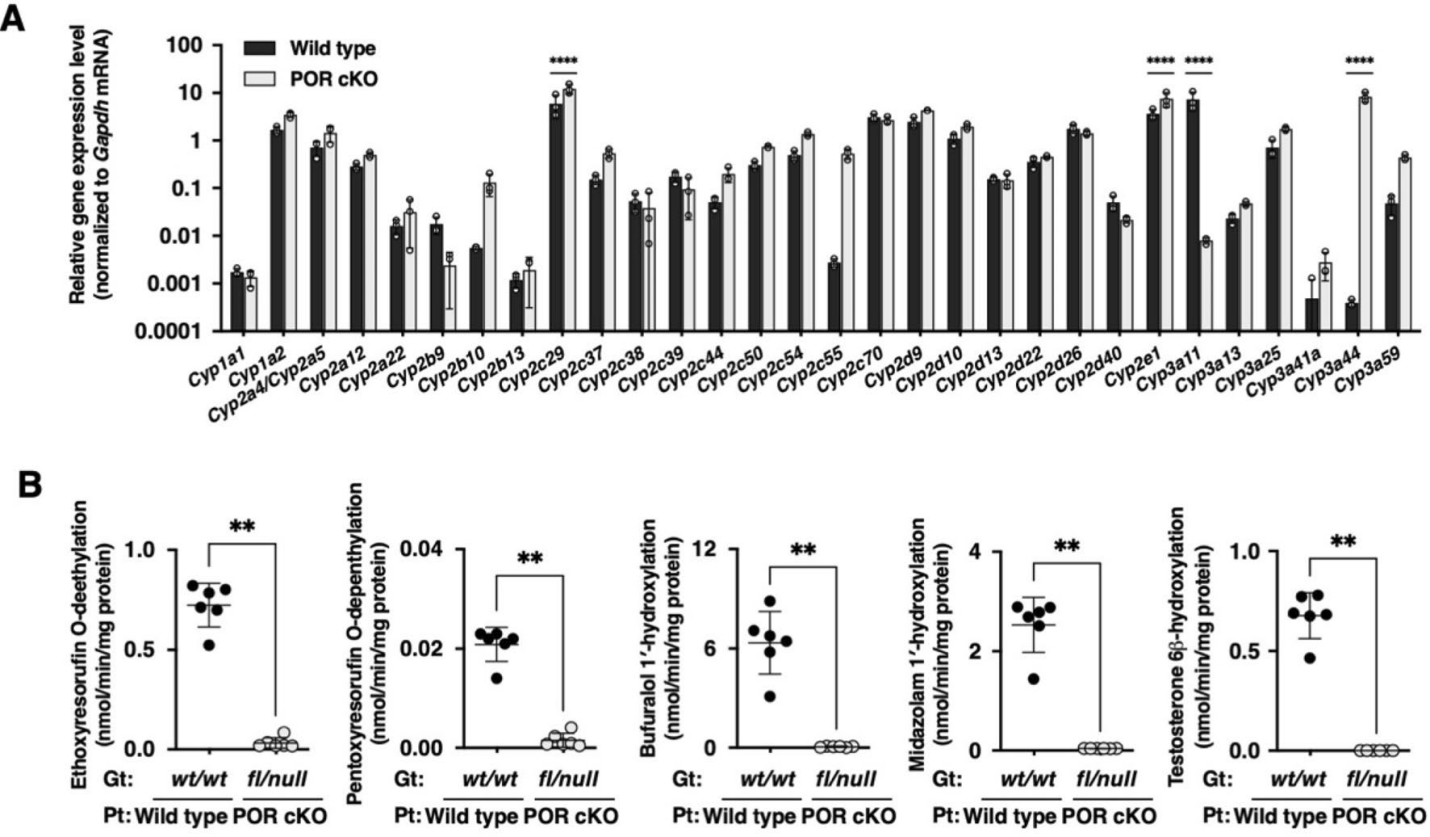
Characterization of POR-knockout phenotype in POR cKO mice. (**A**) Gene expression profiling of livers from wild-type and POR cKO mice. The mRNA expression levels of mouse *P450* genes in livers from wild-type (n = 3) and POR cKO mice (n = 3) were measured via qRT-PCR. Data are presented as the mean ± SD. Statistically significant difference when compared with that in wild-type (TK-NOG) mice; ****p < 0.0001. (**B**) Drug-metabolizing activity in liver microsomes from wild-type and POR cKO mice. Ethoxyresorufin (2 μM), pentoxyresorufin (10 μM), bufuralol (100 μM), midazolam (100 μM), and testosterone (100 μM) were incubated with liver microsomes from wild-type and POR cKO mice (0.50 mg/mL) at 37 °C for 10–20 min. Statistically significant difference when compared with that in wild-type (TK-NOG) mice; **p < 0.01.

We established a conditional knockout mouse strain for the P450 oxidoreductase (*Por*) gene for a humanized liver mouse model. To conditionally delete floxed exons 5–15 of the *Por* gene, two *Flox* sequences, and *Flpo* recombinase gene were introduced to NOG-ES cells by two-step homologous recombination (Fig. 1 and Supplementary Fig. 1). We obtained one chimeric male that exhibited germline transmission of the *Por*^*Flox*^ and *Cyp3a11*^*tm1(Flpo)*^ alleles (Supplementary Fig. 2D). The progeny was used for interbreeding to obtain *Por*^*Flox*^* Cyp3a11*^*tm1(Flpo)*^ homozygotes. *Por*^*Flox*^ homozygotes (*fl/fl*) were obtained through interbreeding with *Por*^*Flox*^ heterozygotes (*fl/wt*) (Fig. 2A). POR protein and gene expression as well as enzymatic activity in the liver and small intestine were examined. The POR conditional knockout (POR cKO) mice with genotype *fl/fl* did not exhibit any knockout phenotype, as determined through immunoblot analysis, qRT-PCR, and enzymatic assays (Fig. 2B,C). Furthermore, immunohistochemical analysis with an anti-POR antibody revealed that some hepatocytes successfully lost POR protein expression (Fig. 2D *middle panel, arrow-heads*), but most hepatocytes expressed POR protein normally. These results indicated that the conditional knockout strategy in POR cKO mice was not fully functional in the *fl/fl* genotype. To ensure that the floxed *Por* gene on both alleles was deleted, the floxed *Por* gene on one allele had been deleted in advance. Mice with the *Por* floxed allele and null allele (abridged name: POR cKO mice) were produced by mating female *Por* floxed homozygotes and male *Por* null heterozygotes. In contrast to POR cKO mice with the *fl/fl* genotype, *fl/null* mice, with one pre-deleted floxed allele, exhibited nearly complete loss of POR function caused by low POR protein and gene expression (Fig. 2B,C). A remarkable reduction in the number of POR-expressing cells was achieved in the livers of POR cKO *fl/null* mice (Fig. 2D *bottom panel*). We used cKO *fl/null* mice as the POR conditional knockout (POR cKO) mouse model in further studies. Hepatic gene expression levels of 30 mouse Cyp enzymes were compared between wild-type and POR cKO mice. Expression of the mouse *Cyp* genes was clearly altered for 18 out of 30 genes in POR cKO mice (Fig. 3A): 15 cytochromes had upregulated expression (< twofold) and 3 cytochromes had downregulated expression (> 0.5-fold). The differences between wild-type and POR cKO mice in three upregulated genes (*Cyp2c29*, *Cyp2e1*, and *Cyp3a44*) and one downregulated gene (*Cyp3a11*) were statistically significant by multiple Mann–Whitney U test. CYP1A, CYP2A, CYP2B, CYP2C, CYP2E, and CYP3A protein levels in liver microsomes from POR cKO *fl/null* mice were higher than those in wild-type and POR cKO *fl/fl* mice (Supplementary Fig. 3). We measured ethoxyresorufin *O*-deethylation, pentoxyresorufin *O*-depenthylation, bufuralol 1′-hydroxylation, midazolam 1′-hydroxylation, and testosterone 6β-hydroxylation in liver microsomes from wild-type and POR cKO mice. All metabolic activities were much lower in the liver microsomes of Por cKO mice than in those of wild-type mice (Fig. 3B). Small intestine *Por* gene expression levels in POR cKO mice of the *fl/null* genotype were one-third lower than those in the wild-type mouse small intestine (Supplementary Fig. 4A). However, cytochrome c reduction activity in the *fl/null* genotype exhibited no significant differences compared with wild-type mice (Supplementary Fig. 4B). Immunohistochemical staining for POR in villi of the small intestine revealed a slight difference (Supplementary Fig. 4C). Furthermore, there was no statistically significant difference in the metabolic activity of small intestinal microsomes between POR cKO mice of the *fl/null* genotype and wild-type mice (Supplementary Fig. 4D).

Next, we investigated the in vivo pharmacokinetics of cytochrome P450 probes (caffeine, racemic warfarin, omeprazole, metoprolol, and midazolam) in wild-type (n = 5) and POR cKO (n = 9) mice after single oral administration at a dose of 1.0 mg/kg. The maximum plasma concentration (C_max_) and the area under the concentration–time curve (AUC_0-inf_) values for the five P450 probes were much higher in POR cKO mice than in wild-type mice (Table 1, Supplementary Fig. 5A–E).

**Table 1:** Pharmacokinetic parameters for caffeine, warfarin, omeprazole, metoprolol, and midazolam in wild-type mice and POR cKO mice after single simultaneous oral administration (1.0 mg/kg each).

Drug	Mouse	C_max_ (ng/mL)	AUC_0-inf_ (µg h/mL)	AUC ratio^a^
Caffeine	Wild-type mice	630 ± 160	2200 ± 250	–
	POR cKO mice	2100 ± 400***	23,000 ± 7000***	10
Warfarin	Wild-type mice	2100 ± 300	29,000 ± 3000	–
	POR cKO mice	4600 ± 1500***	150,000 ± 70,000***	5.2
Omeprazole	Wild-type mice	3.4 ± 1.3	6.6 ± 2.4	–
	POR cKO mice	78 ± 61**	76 ± 59**	12
Metoprolol	Wild-type mice	3.5 ± 1.2	3.6 ± 0.5	–
	POR cKO mice	130 ± 40***	190 ± 20***	53
Midazolam	Wild-type mice	4.7 ± 2.3	6.9 ± 2.6	–
	POR cKO mice	110 ± 45***	810 ± 450***	117

^a^Ratios of AUC_0-inf_ in POR cKO mice to that in wild-type mice. C_max_ and AUC_0-inf_ represent the mean ± SD of six animals (wild-type mice) and nine animals (POR cKO mice). **p < 0.01 and ***p < 0.001 vs. wild-type mice.

### Drug metabolism in POR conditional knockout mice with humanized livers

**Figure 4:**
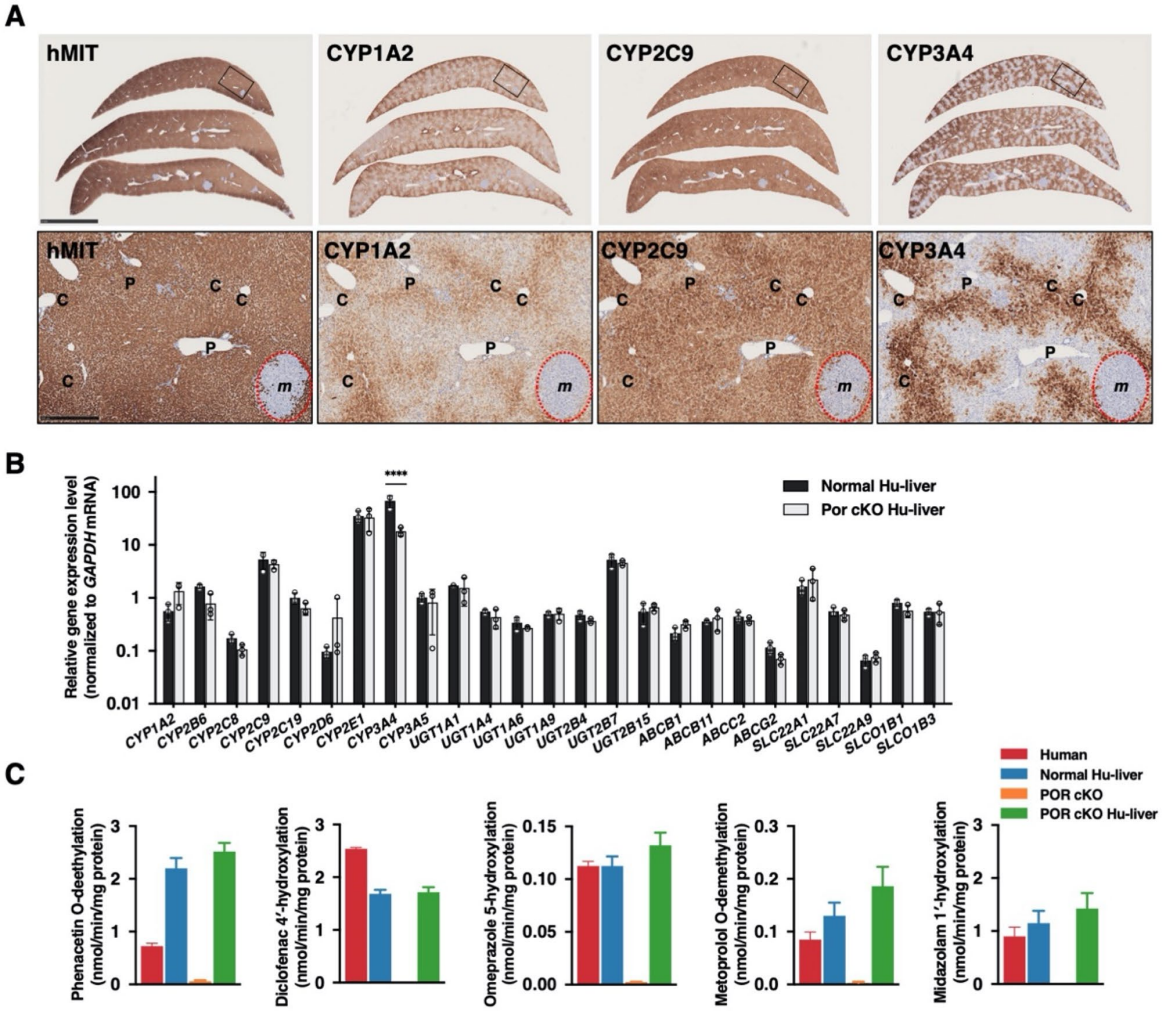
Characterization of POR cKO humanized liver mice. (**A**) Immunohistochemical staining of human Mitochondria (hMIT), CYP1A2, CYP2C9, and CYP3A4 proteins in liver tissue from POR cKO humanized liver mice. Rectangular area on top panel was enlarged in lower panel. Dotted circles indicate the region comprising mouse hepatocytes (*m*). P, portal tract, C, central vein. Scale bar, 5 mm (*top panel*) and 500 μm (*lower panel*). (**B**) Expression level of human pharmacokinetics-related genes in the liver from humanized liver TK-NOG (Normal Hu-liver) mice (n = 3) and POR cKO humanized liver (POR cKO Hu-liver) mice (n = 3) were measured via qRT-PCR. Data are presented as the mean ± SD. Statistically significant difference when compared with that in normal-Hu-liver mice; ****p < 0.0001. (**C**) Drug-metabolizing activity in liver microsomes from TK-NOG humanized liver mice (Normal Hu-liver), POR cKO mice, POR cKO humanized liver mice (POR cKO Hu-liver), and humans. Phenacetin (50 μM), diclofenac (40 μM), omeprazole (10 μM), metoprolol (5 μM), and midazolam (5 μM) were incubated with pooled liver microsomes (0.20 mg/mL) from normal Hu-liver mice (n = 4), POR cKO mice (n = 5), POR cKO Hu-liver mice (n = 4), and humans (n = 50) at 37 °C for 10–20 min. Data are presented as the mean ± SD.

We successfully humanized the livers of POR cKO mice through the transplantation of human hepatocytes (POR cKO Hu-liver). Most hepatocytes in POR cKO Hu-liver mice were replaced by human hepatocytes stained with an anti-human mitochondria antibody (Fig. 4A). The portal triad, consisting of the portal vein, hepatic artery, and intrahepatic bile duct, was formed in the periphery of the lobule in POR cKO Hu-liver mice. We then examined consecutive thin tissue sections prepared from POR cKO Hu-liver mice with specific antibodies against human P450s. CYP1A2 and CYP3A4 proteins were localized to the perivenous regions. By contrast, CYP2C9 proteins were distributed uniformly across hepatic lobules (Fig. 4A). Immunostaining results were consistent with those of the adult human liver described in our previous report^14^. The expression levels of 25 major pharmacokinetics-related genes were compared between TK-NOG humanized liver (Normal Hu-liver) and POR cKO Hu-liver mice. Most of the genes (24 of 25 genes) were expressed at comparable levels in the two groups of mice. Only the *CYP3A4* gene had significantly lower expression in POR cKO Hu-liver mice than Normal Hu-liver mice (Fig. 4B). To characterize metabolic function in the POR cKO Hu-liver, we measured phenacetine *O*-deethylation, diclofenac 4′-hydroxylation, omeprazole 5-hydroxylation, metoprolol *O*-deethylation, and midazolam 1′-hydroxylation in liver microsomes from POR cKO Hu-liver mice. The drug-metabolizing enzymes involved in these reactions were scarce or absent within liver microsomes from POR cKO mice. The engraftment of humanized livers in POR cKO mice showed comparable or higher drug-metabolizing activities than those in normal Hu-liver and human liver microsomes (Fig. 4C).

**Figure 5:**
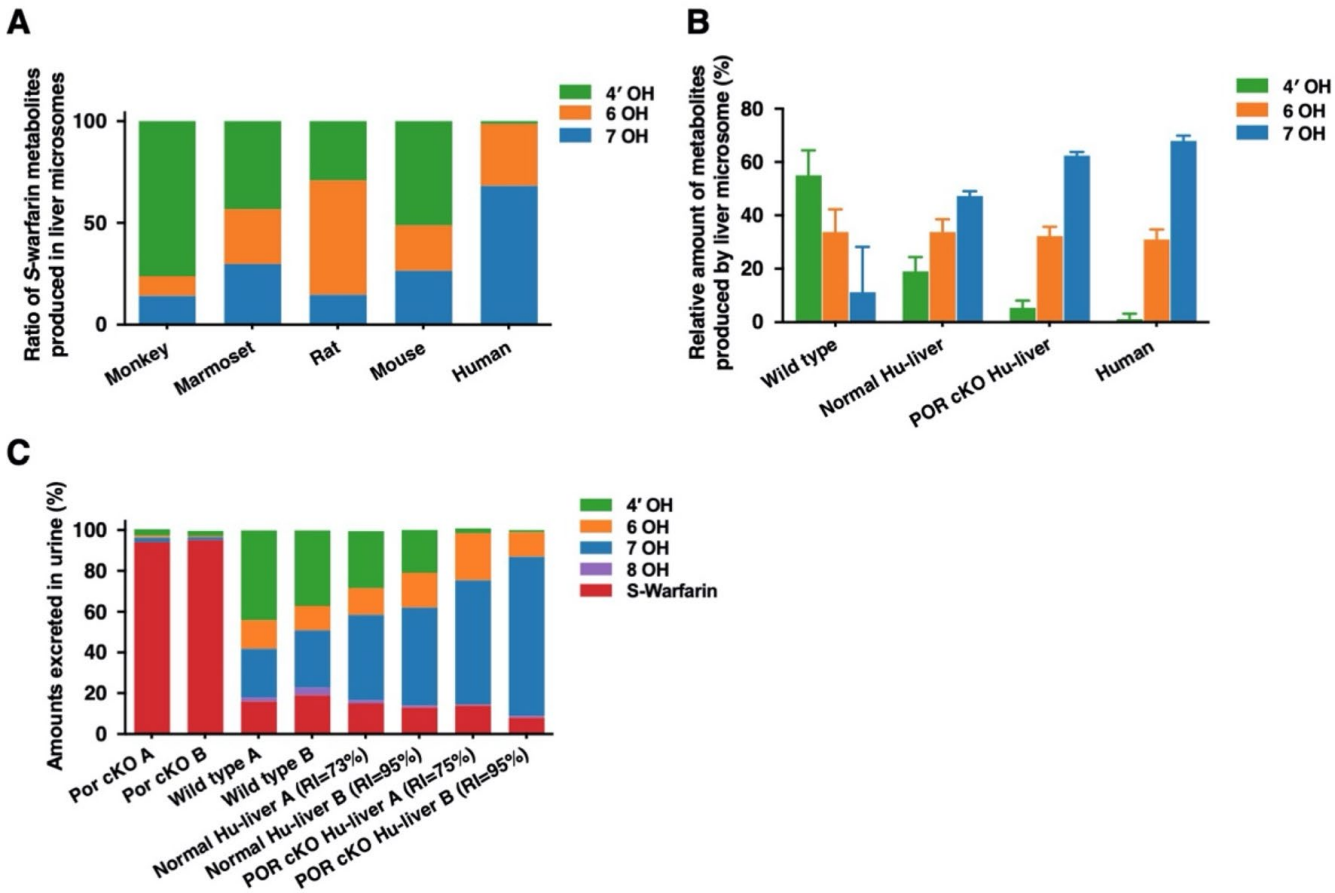
Characterization of *S*-warfarin metabolism in POR cKO humanized liver mice. (**A**) Species-dependent regioselective *S*-warfarin hydroxylation in various animal liver microsomes. *S*-warfarin (10 μM) was incubated with pooled liver microsomes (0.20 mg/mL) at 37 °C for 20 min. (**B**) *S*-warfarin metabolism in pooled liver microsomes from wild-type (six males), humanized liver TK-NOG (Normal Hu-liver, four males) mice, POR cKO humanized liver (POR cKO Hu-liver, four males) mice, and humans. *S*-warfarin (10 μM) were incubated with the liver microsomes (0.20 mg/mL) at 37 °C for 20 min. (**C**) Cumulative urinary excretions of *S*-warfarin and its metabolites for 72 h after intravenous administration (4.0 mg/kg) in wild-type, POR cKO, Normal Hu-liver, and POR cKO Hu-liver mice. The 4′-, 6-, 7-, 8-, and 10-hydroxywarfarin were measured for urine samples treated with β-glucuronidase. The data presented are from two animals.

**Figure 6:**
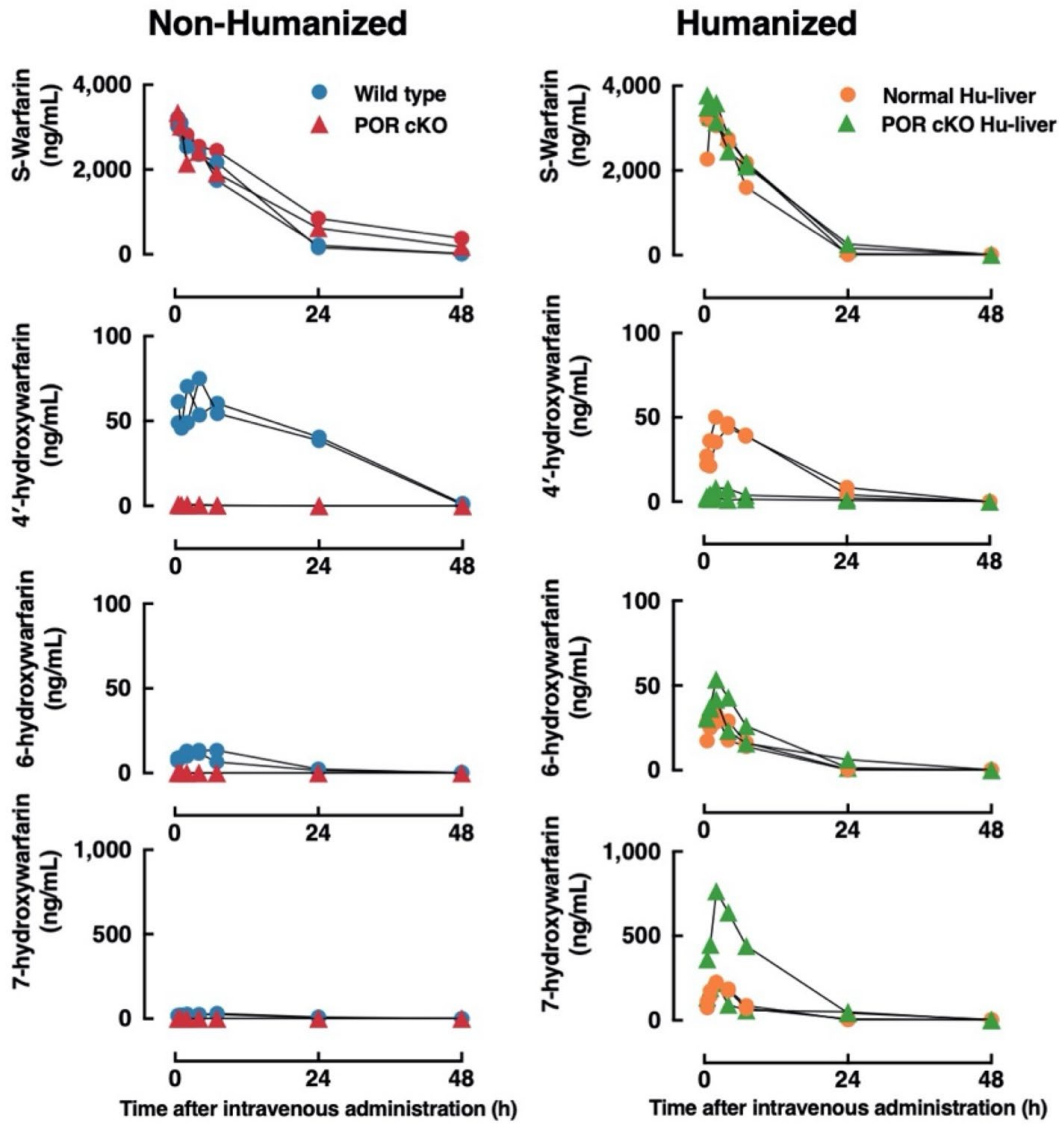
Plasma concentration of *S*-warfarin and its hydroxy metabolites after the intravenous administration of *S*-warfarin (4.0 mg/kg) in non-humanized liver mice and humanized liver mice. The plasma concentrations of *S*-warfarin, 4′-hydroxywarfarin, 6-hydroxywarfarin, and 7-hydroxywarfarin in wild-type (circle) or POR cKO (triangle) mice with non-humanized or humanized livers were measured at 0.5, 1, 2, 4, 7, 24, 48, and 72 h after the intravenous administration of *S*-warfarin (4.0 mg/kg). Each group consists of two mice. The levels of 8- and 10-hydroxywarfarin were below the detection limit in all samples.

Species-dependent regioselective *S*-warfarin hydroxylation was observed in various liver microsomes (Fig. 5A). Liver microsomes from wild-type mice preferentially catalyzed the *S*-4′-hydroxylation of *S*-warfarin, while *S*-7-hydroxylation of *S*-warfarin was the major metabolite in human liver or normal Hu-liver microsomes (Fig. 5B). Given the differences in *S*-warfarin metabolism observed in vitro, we further analyzed the pharmacokinetics and metabolism of *S*-warfarin in POR cKO Hu-liver mice. The plasma concentration versus time curves of *S*-warfarin and its metabolites (*S*-4′-/6-/7-hydroxywarfarin) after a single intravenous administration of *S*-warfarin to wild-type and POR cKO mice with or without humanized livers are shown in Fig. 6. The AUC_0-inf_ for *S*-4′-hydroxywarfarin was lower in POR cKO Hu-liver mice than in normal Hu-liver mice, whereas AUC_0-inf_ for *S*-6-/7-hydroxywarfarin was higher in POR cKO Hu-liver mice than in normal Hu-liver mice (Table 2). The composition of *S*-4′-/6-/7-/8-/10-hydroxywarfarin in β-glucuronidase-treated urine was compared between wild-type and POR cKO mice with or without humanized livers (Fig. 5C). The most abundant metabolite in the urine of POR cKO Hu-liver mice after administration of *S*-warfarin was *S*-7-hydroxywarfarin, followed by *S*-6-hydroxywarfarin, *S*-4′-hydroxywarfarin and 8-hydroxywarfarin (61%, 78%; 12%, 23%; 2.5%, 1.2% and 0.37%, 0.98% of excreted amounts in each urine sample, respectively) (Fig. 5C, Supplementary Table 6). Notably, the excreted amount of *S*-4′-hydroxywarfarin in urine was much lower in POR cKO Hu-liver mice than in normal Hu-liver mice, reflecting the differences in metabolic properties between POR-expressing or POR-deleted livers. In contrast to urine, *S*-warfarin and its hydroxy metabolites could not be detected in the feces.

**Table 2:** AUC_0-inf_ of metabolites after single intravenous administration of *S*-warfarin at 4.0 mg/kg to wild-type, POR cKO, Normal Hu-liver, and POR cKO Hu-liver mice.

Metabolite	Mouse	AUC_0-inf_ (μg h/mL)	AUC ratio^a^
4′-Hydroxywarfarin	Wild-type mouse A	1700	–
	Wild-type mouse B	1800	–
	POR cKO mouse A	3.3	0.0019
	POR cKO mouse B	6.3	0.0036
	Normal Hu-liver mouse A	700	0.4000
	Normal Hu-liver mouse B	750	0.4300
	POR cKO Hu-liver mouse A	68	0.0390
	POR cKO Hu-liver mouse B	140	0.0800
6-Hydroxywarfarin	Wild-type mouse A	240	–
	Wild-type mouse B	170	–
	POR cKO mouse A	0.18	0.00088
	POR cKO mouse B	0.28	0.00140
	Normal Hu-liver mouse A	280	1.4
	Normal Hu-liver mouse B	320	1.6
	POR cKO Hu-liver mouse A	450	2.2
	POR cKO Hu-liver mouse B	530	2.6
7-Hydroxywarfarin	Wild-type mouse A	620	–
	Wild-type mouse B	530	–
	POR cKO mouse A	3.3	0.0057
	POR cKO mouse B	3.8	0.0066
	Normal Hu-liver mouse A	1900	3.3
	Normal Hu-liver mouse B	2000	3.5
	POR cKO Hu-liver mouse A	2400	4.2
	POR cKO Hu-liver mouse B	8700	15.0

^a^Ratios of AUC_0-inf_ in POR cKO mice, humanized liver TK-NOG mice (Normal Hu-liver), and POR cKO humanized liver (POR cKO Hu-liver) mice compared to those in wild-type mice (mean value of two animals). Normal Hu-liver mouse A and B (human Albumin: 6.5 and 6.6 mg/mL, Cholinesterase activity 255 and 425 U/L, Replacement Index 72.5% and > 95% estimated with Cholinesterase activity), POR cKO Hu-liver mouse A and B (human Albumin: 7.6 and 8.3 mg/mL, Cholinesterase activity 265 and 470 U/L, Replacement Index 75.1% and > 95% estimated with Cholinesterase activity).

## Discussion

In the present work, we generated an improved humanized mouse model for the predictive study of human drug metabolism with minimal interference from murine hepatic P450s. A comparison of in vivo* S*-warfarin metabolism in TK-NOG humanized liver (normal Hu-liver) mice and POR cKO humanized liver (POR cKO Hu-liver) mice was performed. The production of *S*-4′-hydroxywarfarin (a metabolite predominantly produced in mouse hepatocytes) was lower in POR cKO Hu-liver mice than in normal Hu-liver mice. By contrast, *S*-7-hydroxywarfarin (a metabolite preferentially produced in human hepatocytes) was more abundant in the urine of POR cKO Hu-liver mice, which was closer to the composition of *S*-warfarin metabolites in human urine after a single oral dose of *racemic*-warfarin, as previously reported^15^. The major urinary metabolite of *S*-warfarin in humans after a single oral dose of *racemic*-warfarin is reported to be *S*-7-hydroxywarfarin and with no *S*-4′-hydroxywarfarin detected^16^. Inoue et al. investigated *S*-warfarin metabolic pathways with chimeric mice (PXB®) after a single oral dose of *S*-warfarin and reported that urinary levels of 7-hydroxywarfarin (including 8-hydroxywarfarin), 4′-hydroxywarfarin, 6-hydroxywarfarin, and unchanged *S*-warfarin were 15.6%, 12.4%, 6.5%, and 4.8%, respectively^17^. The ratio of the major metabolite in humans (7-hydroxywarfarin) to the major metabolite in mice (4′-hydroxywarfarin) was 1.26 in chimeric mice and 0.39 in control mice (uPA^+/+^, SCID ^wt/wt^), approximately threefold higher in chimeric mice. They reported that the disposition of *S*-warfarin in chimeric mice was similar to that reported for *S*-warfarin in humans. However, Inoue et al. mentioned that because 4′-hydroxywarfarin is not detected in urine in humans^16^, it is still present 4′-hydroxywarfarin in chimeric mice at 12% (at 18% in control mice); therefore, the majority of 4′-hydroxywarfarin detected in chimeric mice may have been host mouse-derived. In the present study, we report that the urinary metabolites of *S*-warfarin in normal Hu-liver mice after a single intravenous dose of *S*-warfarin were *S*-7-hydroxywarfarin (42.0%, 48.0%), followed by *S*-4′-hydroxywarfarin (28.0%, 21.0%), *S*-6-hydroxywarfarin (13.0%, 17.0%), and unchanged *S*-warfarin (15.0%, 13.0%). The ratio of *S*-7-hydroxywarfarin to *S*-4′-hydroxywarfarin in normal Hu-liver mice was 1.50 and 2.29, and in control mice (wild type), it was 0.55 and 0.76. These ratios were similar to those reported by Inoue et al.^17^. These results suggested that even in highly chimeric Hu-liver mice, a small amount of residual mouse hepatocytes influence the metabolic activity as a noise, obscuring the original human metabolic profile. In POR cKO Hu-liver mice after a single intravenous dose of *S*-warfarin, urinary metabolites were *S*-7-hydroxywarfarin (61.0%, 78.0%), followed by *S*-6-hydroxywarfarin (23.0%, 12.0%), unchanged *S*-warfarin (14.0%, 7.9%), and *S*-4′-hydroxywarfarin (2.5%, 1.2%). The order of the content of metabolites was similar to that in humans^16^. The ratios of *S*-7-hydroxywarfarin to *S*-4′-hydroxywarfarin were 24.4 and 65.0, which were much higher than those in conventional human liver chimeric mice. These results clearly show that the POR cKO Hu-liver model seems to have a closer disposition to humans than the other models. Furthermore, the removal of residual mouse hepatocyte metabolism also resulted in an increase in the production of metabolites that should have appeared (reduced false-negative results). In the case of *S*-warfarin, the *S*-4′-hydroxywarfarin, which should not appear in chimeric mice, is a false-positive product generated by the remaining mouse hepatocytes, and the *S*-7-hydroxywarfarin is a false-negative product whose production was greatly suppressed by the presence of the remaining mouse hepatocytes. These results indicate that POR cKO Hu-liver mice would be a highly effective in vivo model for predicting drug metabolism in humans when the metabolic activities of residual mouse hepatocytes are concerned. The advantage of this model is not simply the ability to detect metabolites predominantly produced in humans, but the ability to make true human metabolism visible by eliminating mouse metabolism derived from remaining mouse hepatocytes.

Compared to the normal Hu-liver mouse model, POR cKO Hu-liver mice could be useful for predicting drug metabolism and pharmacokinetics in humans, especially with regard to P450 substrates that are predominantly metabolized in the liver. However, the extrahepatic tissues of humanized liver mice are derived from mice. Drug-metabolizing activities in extrahepatic tissues vary among species. The kinetic profile of midazolam oxidation in liver and intestine microsomes from mice and humans are similar, suggestive of high similarity in intestinal CYP3A function between mice and humans^18^. In contrast, 7-ethoxycoumarin *O*-deethylation (CYP1, 2B, 2D, and 3A-catalytic reactions) and pentoxyresorufin *O*-depenthylation (CYP2B-catalytic reaction) activities in the lungs were largely different between humans and mice^19^. Therefore, POR cKO Hu-liver mice should be utilized for predicting drug metabolism and pharmacokinetics in humans, considering the interspecies differences in extrahepatic drug-metabolizing activity, such as the small intestine, kidneys, and lungs.

The small intestine plays an important role in the first-pass metabolism of certain drugs. The expression level of *Por* mRNA in the small intestine was lower in POR cKO mice than in wild-type mice (Supplementary Fig. 4A). Since mRNA expression is decreased, the amount of newly synthesized POR protein is expected to decrease. On the contrary, already synthesized POR proteins will exist and function until they are degraded, or the cells are destroyed. Actually, no significant differences in POR activity and protein were observed in either mouse (Supplementary Fig. 4B,C). The turnover of small intestinal epithelial cells is not considered to be long. In fact, the turnover time of intestinal epithelial cells was measured by BrdU labeling to be approximately 3 days (Supplementary Fig. 4E). Within this short survival time, we speculate that it is difficult to completely delete the floxed *Por* gene and to completely degrade the already synthesized POR protein.

POR cKO mice exhibited an enlarged fatty liver (Fig. 2D *bottom panel*), which might account for the suppression of genes involved in fatty acid metabolism. Indeed, six genes of the fatty acid oxidation pathway (*Cpt2*, *Acadvl*, *Acadl*, *Hadhb*, *Ehhadh*, and *Dci*) were suppressed in the livers of other hepatic POR-null mice, indicating reduced fatty acid utilization^20^. Upregulation of *Cyp2b10* and *Cyp2c55* mRNA levels in POR cKO mouse livers may be due to the induction of the same genes following activation of the constitutive androstane receptor in hepatic POR-null mouse livers (Fig. 3A)^20^. The upregulation of CYP1A, CYP2A, CYP2C, CYP2E, and CYP3A proteins in POR cKO mice was consistent with the characteristics of other POR-null mice^21^. Overall, the POR cKO mice exhibited phenotypic characteristics of the traditional POR-null line.

Barzi et al. reported the functional superiority of the conditional knockout of the NADPH-P450 oxidoreductase gene combined with FRG (*Fah*^−/−^/*Rag2*^−/−^/*Il2rg*^−/−^) humanized liver mice (PIRF mice)^13^. However, the PIRF mouse model is a slightly complicated system in which *Cre* recombinase is expressed by adenoviral transduction to conditionally delete the *Por* gene in the liver. On the contrary, PIRF mice have the advantage that *Cre* recombinase expression can be controlled by infection with adenovirus vectors, allowing direct comparison of its effects before and after *Por* gene deletion. In this regard, the *Flp* recombinase gene in POR cKO mouse is constitutively expressed under the promoter of the *Cyp3a11* gene which is highly expressed in the liver and small intestine; therefore, it is not possible to make a direct comparison before and after *Por* gene deletion. The advantage of the POR cKO mice we established is that (1) no treatment is required to delete the *Por* gene, (2) the knock-in of the *Flp* gene into the *Cyp3a11* gene should result in loss of function of the *Cyp3a11* gene in addition to the *Por* gene. This effect was confirmed by a significant decrease in *Cyp3a11* mRNA expression (Fig. 3A) and loss of CYP3A enzyme activity (hydroxylation of midazolam and testosterone) (Fig. 3B).

In conclusion, the residue mouse hepatic P450 activity compromises the accuracy of the traditional humanized liver model for the predictive study of human drug metabolism, which can lead to poor prediction of the efficacy and toxicity of a drug candidate. In this study, we successfully established a novel POR-null humanized liver TK-NOG mouse model for drug metabolism studies. This model exhibited a reduction in mouse hepatic P450 activity resulting from liver-specific *Por* deletion. The minimal interference of drug-metabolizing activity in residual mouse hepatocytes represents a major advantage of this novel model over the traditional humanized liver model. Further validation studies with more compounds are needed to confirm the usefulness of the POR cKO Hu-liver mice. Overall, the POR cKO Hu-liver mouse is expected to become the preferred platform for the study of human drug metabolism and pharmacokinetics.

## Methods

### Generation of P450 oxidoreductase conditional knockout mice

All animals used in this study were maintained in the Central Institute for Experimental Animals (CIEA) under specific pathogen-free conditions. All experiments were performed in accordance with institutional guidelines (090014 and 17017A), which were approved by the Animal Experimentation Committee of CIEA. The institutional guidelines are in compliance with the ARRIVE guidelines. Two-step homologous recombination strategies using NOG-ES cells (Supplementary Materials and Methods) for conditional knockout of the P450 oxidoreductase (*Por*) gene in the liver and small intestine are illustrated in Supplementary Fig. 1. To achieve conditional deletion of floxed *Por* exons 5–15 in the liver and small intestine, *Flpo* recombinase gene was expressed under the *Cyp3a11* promoter, which functions effectively in the liver and small intestine, by targeted transgenesis with homologous recombination (knock-in). One chimeric male exhibited germline transmission of the *Por*^*Flox*^ and *Cyp3a11*^*tm1(Flpo)*^ alleles (Supplementary Fig. 2D). This conditional knockout mouse strain was assigned the following genetic designation: NOG-*Por*^*tm1*^*Cyp3a11*^*tm1*(*Flpo*)*Jic*^ (formally NOD.Cg-*Prkdc*^*scid*^* Il2rg*^*tm1Sug*^* Por*^*tm1*^* Cyp3a11*^*tm1*(*Flpo*)*Jic*^/Jic). To ensure that the floxed *Por* gene on both alleles was deleted, the floxed *Por* gene on one allele had been deleted in advance. Mice with the *Por* floxed allele and null allele (abridged name: POR cKO mice) were produced by mating female *Por* floxed homozygotes and male *Por* null heterozygotes. The *Por* genotypes were determined using multiplex PCR using the following primers: forward 5′-TTATGTTGAGGCTCTTAGTAACTCG-3′ (mPor-F1) and reverse 5′-AAAGGTGGGTCCAGTCCCTCTTGC-3′ (mPor-R2del) and 5′-TCAGTGACCTTACATGGAAGCTCG-3′ (mPor-R2wild). The sizes of amplicons derived from the wild-type allele (*wt*), floxed allele (*fl*), and null allele (*null*) were 399 bp, 461 bp, and 810 bp, respectively.

### Preparation of humanized liver chimeric mice

Recipients for human hepatocytes were produced by crossbreeding POR cKO mice and TK-NOG mice, a hepatic injury model. The methods for liver humanization employed in the TK-NOG model have been described previously^22^. Briefly, the mice were given 0.06 mg/mL valganciclovir for 3 days to ablate hepatocytes expressing the HSV-TK transgene. Human hepatocytes (1.0 × 10^6^ cells/mouse) were injected into the spleens of liver-injured mice. Cryopreserved human hepatocytes (12-year-old Caucasian female [donor A] and 30-year-old African American female [donor B]) were provided by Lonza Walkersville Inc. (Walkersville, MD, USA). The replacement index of the humanized liver was evaluated by measuring the blood levels of human albumin with a human albumin ELISA quantitation kit (Bethyl Laboratories, Montgomery, TX, USA) or butyrylcholinesterase (ChE) activity (Fuji Dri-Chem 7000; Fuji Photo Film, Tokyo, Japan).

### Quantitative RT-PCR (qRT-PCR)

Total RNA was prepared using an RNeasy Mini Kit (Qiagen, Valencia, CA, USA). First-strand cDNA was reverse-transcribed from hepatic total RNA using a High-Capacity cDNA Reverse Transcription Kit (Thermo Fisher Scientific, Waltham, MA, USA). Quantitative RT-PCR was performed using TaqMan gene expression assays (Thermo Fisher Scientific) on a 7500 Fast Real-Time PCR System (Applied Biosystems, Foster City, CA, USA). The TaqMan probes used for mouse and human genes are listed on Supplementary Tables 4 and 5, respectively. Expression levels of human and mouse genes were normalized to that of GAPDH from humans (Hs99999905_m1) and mice (Mm99999915_g1), respectively.

### In vitro enzyme assay using tissue microsomes

The preparation of microsomes from the liver and/or small intestine has been described previously^14^. The NADPH-cytochrome c reduction activity was determined as previously described^23^. Ethoxyresorufin *O*-deethylation, pentoxyresorufin *O*-depenthylation, bufuralol 1′-hydroxylation, and testosterone 6b-hydroxylation were measured using high-performance liquid chromatography (HPLC) with fluorescence or an ultraviolet detector, as previously described, with slight modifications^23,24^. Phenacetine *O*-deethylation, diclofenac 4′-hydroxylation, omeprazole 5-hydroxylation, metoprolol *O*-demethylation, midazolam 1′-hydroxylation, and *S*-warfarin hydroxylation in liver microsomes were measured using a liquid chromatography–tandem mass spectrometry (LC–MS/MS) system. The reaction mixture (250 μL) contained liver microsomes (0.2 mg/mL protein) and any one substrate in 100 mM potassium phosphate buffer (pH 7.4). Following pre-incubation at 37 °C for 3 min, reactions were initiated through the addition of an NADPH-generating system (0.25 mM NADP^+^, 2.5 mM glucose 6-phosphate, and 0.25 unit/mL glucose-6-phosphate dehydrogenase). After incubation at 37 °C for 10 min (5 μM metoprolol and 5 μM midazolam) or 20 min (50 μM phenacetin, 40 μM diclofenac, 10 μM omeprazole, and 10 μM *S*-warfarin), the reactions were terminated by the addition of 250 μL of ice-cold acetonitrile containing 8-cyclopentyl-1,3-dimethylxanthine (internal standard). All samples were centrifuged at 20,000×*g* for 10 min. Two microliters of the supernatant was injected into the LC–MS/MS system.

### In vivo drug metabolism study

To evaluate the effect of POR deletion, five P450 substrates (caffein, omeprazole, midazolam, warfarin, and metoprolol) were orally administered to wild-type and POR cKO mice. Dosing solutions were prepared as five-drug composites (cassette dosing) at doses of 1.0 mg/kg each. Plasma samples were collected from control and humanized liver mice 0.5, 1, 2, 4, 7, and 24 h after P450 substrate cocktail administration.

To evaluate the effect of *Por* deletion in humanized liver mice, *S*-warfarin was intravenously administered to TK-NOG (wild type in this study), POR cKO, humanized liver, and POR cKO humanized liver mice at doses of 0.20 mg/kg each. Plasma samples were collected at 0.5, 1, 2, 4, 7, 24, 48, and 72 h after *S*-warfarin administration. Accumulated urinary and fecal samples (0–72 h) were collected from all mice. Five volumes of methanol were added to the feces, followed by homogenization and centrifugation at 20,000×*g* for 10 min to collect the supernatant as an extract. Plasma, urine, and fecal solutions (5 µL) were deproteinized by adding 20 µL of acetonitrile containing 8-cyclopentyl-1,3-dimethylxanthine (internal standard) and centrifuged at 20,000×*g* for 10 min at 4 °C. Urine samples were incubated with 0.2 M sodium acetate buffer (pH 5.0) and with solid β-glucuronidase (Helix pomatia, 20,000 units/mL; Wako Pure Chemical Industries) at 37 °C for 18 h.

### LC–MS/MS analysis

Measurements of unchanged drugs and their metabolite concentrations in the supernatant of in vitro reaction samples, plasma, urine, and fecal solutions were performed on an API QTRAP 5500 triple quadrupole mass spectrometer (AB SCIEX, Foster, CA, USA) coupled with a Nexera ultra-high-performance liquid chromatography system (Shimadzu, Kyoto, Japan). The chromatographic separation was performed on a YMC-Triart C18 column (3 μm, 3.0 mm × 100 mm; YMC, Kyoto, Japan), and the column temperature was set at 40 °C. Gradient elution was achieved in water with 0.1% formic acid (solvent A) and methanol with 0.1% formic acid (solvent B) as the mobile phase, per the following procedure: 0–12 min, 20–80% B; 12–15 min, 100% B; 15–18 min, 20% B at a flow of 0.3 mL/min. The autosampler was maintained at 10 °C, and the injection volume was 2 μL for analysis. The mass spectrometer was operated in the positive ion electrospray ionization mode, and ions detected in the multiple reaction monitoring mode with a precursor-to-product mass transition (*m/z* precursor ion > product ion) of *m/z* 195.1 > 138.0 for caffeine, *m/z* 309.1 > 163.0 for warfarin, *m/z* 346.1 > 198.1 for omeprazole, *m/z* 268.0 > 116.1 for metoprolol, *m/z* 326.2 > 291.1 for midazolam, *m/z* 152.0 to *m/z* 93.0 for acetaminophen, *m/z* 325.1 > 163.0 for 4′-hydroxywarfarin, *m/z* 325.1 > 179.0 for 6-, 7-, and 8-hydroxywarfarin, *m/z* 325.1 > 251.1 for 10-hydroxywarfarin, *m/z* 362.1 > 152.0 for 5-hydroxyomeprazole, *m/z* 254.2 > 177.1 for *O*-demethylmetoprolol, and *m/z* 342.0 > 324.1 for 1′-hydroxymidazolam, and *m/z* 249.1 > 192.2 for 8-cyclopentyl-1,3-dimethylxanthine.

### Immunohistochemistry

Immunohistochemical staining with monoclonal mouse anti-human mitochondria (hMIT) (clone 113-1, Merck Millipore, Burlington, MA, 1:2000), antihuman CYP1A2 (clone 3B8C1, Abcam plc., Cambridge, UK, 1:1000), antihuman CYP2C9 (clone 2C8, LifeSpan Biosciences, Inc., Seattle WA. USA, 1:150), and rabbit antihuman CYP3A4 (clone EPR6202, Abcam Plc., 1:300) antibodies was performed as described previously^14^. Tissues were fixed in 4% (v/v) phosphate-buffered formalin (Mildform 10 NM; Wako Pure Chemical Industries). The sections were autoclaved for 10 min in a target retrieval solution (0.1 M citrate buffer, pH 6.0; 1 mM EDTA, pH 9.0), equilibrated at room temperature for 20 min, and then incubated with an anti-POR (HPA010136, Sigma) primary antibody. Primary antibodies were visualized using amino acid polymer/peroxidase complex-labeled antibodies (Histofine Simple Stain MAX PO [MULTI]; Nichirei Biosciences Inc.) and diaminobenzidine (DAB; Dojindo Laboratories, Kumamoto, Japan) substrate (0.2 mg/mL 3,3-diaminobenzidine tetrahydrochloride in 0.05 M Tris–HCl, pH 7.6, and 0.005% H_2_O_2_). The sections were counterstained with hematoxylin. Images were captured using a digital slide scanner (NanoZoomer S60; Hamamatsu Photonics, KK Hamamatsu, Japan).

### Western blot analysis

Liver microsomes (5–20 μg) were subjected to 10% sodium dodecyl sulfate–polyacrylamide gel electrophoresis and then electrophoretically transferred to a polyvinylidene difluoride membrane (Merck, Darmstadt, Germany). After blocking with 0.5% non-fat milk in Tris-buffered saline (50 mM Tris, 138 mM NaCl, 2.7 mM KCl) containing 0.05% Tween 20 (v/v) at room temperature for 30 min, the membrane was probed with anti-human POR antibodies (1:2000; HPA010136, Sigma), anti-human CYP1A2 antibodies (1:2000; 19936-1-AP, Proteintech, Rosemont, IL, USA), anti-human CYP2A antibodies (1:5000; PAP061, Nosan Corporation, Yokohama, Japan), anti-mouse P450 2b10 antibodies (1:500; AB9916, Merck Millipore), anti-human P450 2C9 antibodies (1:1000; HPA015066, Sigma), anti-rat P450 2D1 antibodies (1:500; BML-CR3210, Enzo Life Sciences, Farmingdale, NY, USA), anti-rat/human CYP2E1 antibodies (1:2000; CR3271, Enzo Life Sciences), anti-rat CYP3A2 antibodies (1:1000; R-PAP 171, Nosan Corporation), or anti-protein disulfide isomerase (PDI) antibodies (1:200; 11245-1-AP, Proteintech) at room temperature for 1 h. The membrane was then incubated with goat anti-rabbit IgG antibodies (1:5000; GE Healthcare, Chicago, IL, USA) at room temperature for 20 min. Reactive bands were visualized using the ECL Prime Western Blotting Detection System (GE Healthcare).

### Statistical analyses

Differences in microsomal drug-metabolizing activities between wild-type and POR cKO mice were assessed using one-way analysis of variance with Dunnett’s post-hoc test and differences in liver drug-metabolizing enzyme gene expression between wild-type and POR cKO mice were assessed using multiple Mann–Whitney U tests in Prism 9 (GraphPad Software, San Diego, CA, USA). *p* values of < 0.05 were considered statistically significant. Pharmacokinetic parameters were calculated using non-compartmental analysis with Phoenix WinNonlin (version 7.0; Certara, Princeton, NJ, USA). The plasma levels of the drug and its metabolites were compared using two-way analysis of variance followed by Bonferroni’s *post-hoc* test in GraphPad Prism.

## Supplementary Information

Supplementary Information.

## Data Availability

The datasets generated during and/or analysed during the current study are available from the corresponding author on reasonable request.
