# Supplementary material for: Humanized liver TK-NOG mice with functional deletion of hepatic murine cytochrome P450s as a model for studying human drug metabolism
Source: Sci Rep. 2022 Sep 1;12:14907. doi: 10.1038/s41598-022-19242-0 (PMC9437039; doi:10.1038/s41598-022-19242-0)
Supplement: Supplementary file 1 — Supplementary Information. [file 41598_2022_19242_MOESM1_ESM.docx]

**Humanized liver TK-NOG mice with functional deletion of hepatic murine cytochrome P450s as a model for studying human drug metabolism**

Shotaro Uehara^1^, Yuichi Iida^2§^, Miyuki Ida-Tanaka^1^, Motohito Goto^3^, Kenji Kawai^4^, Masafumi Yamamoto^5^, Yuichiro Higuchi^1^, Satoshi Ito^6^, Riichi Takahashi^3^, Hidetaka Kamimura^6#^, Mamoru Ito^1^, Hiroshi Yamazaki^7^, Mitsuo Oshimura^2^, Yasuhiro Kazuki^2,8^, and Hiroshi Suemizu^1*^

^1^Liver Engineering Laboratory, Department of Applied Research for Laboratory Animals, Central Institute for Experimental Animals (CIEA), Kawasaki, Japan

^2^Chromosome Engineering Research Center (CERC), Tottori University, Yonago, Japan

^3^Animal Resource & Technical Research Center, CIEA, Kawasaki, Japan

^4^Pathological Analysis Center, CIEA, Kawasaki, Japan

^5^ICLAS Monitoring Center, CIEA, Kawasaki, Japan

^6^ Drug Development Solutions Center, Sekisui Medical Co., Ltd., Ibaraki, Japan

^7^Laboratory of Drug Metabolism and Pharmacokinetics, Showa Pharmaceutical University, Machida, Japan

^8^Department of Chromosome Biomedical Engineering, School of Life Science, Faculty of Medicine, Tottori University, Yonago, Japan

**Present address**

§ Department of Immunology, Shimane University Faculty of Medicine, Izumo, Japan, # Laboratory Animal Research Department, CIEA, Kawasaki, Japan,

^*^**Corresponding author**

Hiroshi Suemizu, Central Institute for Experimental Animals, 3-25-12 Tonomachi, Kawasaki-ku, Kawasaki, 210-0821, Japan. Phone: +81-44-201-8530; Fax: +81-44-201-8541. E-mail: suemizu@ciea.or.jp

**Supplementary Materials and Methods**

***Chemicals and reagents***

Caffeine, warfarin, omeprazole, and midazolam were purchased from Fujifilm Wako Pure Chemical Industries (Osaka, Japan). Metoprolol was obtained from Tokyo Chemical Industry (Tokyo, Japan). Pentoxyresorufin, *S*-warfarin, 5-bromo-2-deoxyuridine (BrdU), and Val-ganciclovir were purchased from Sigma-Aldrich (St. Louis, MO, USA). Ethoxyresorufin, 7-hydroxywarfarin, 5-hydroxyomeprazole, *O*-demethylmetoprolol, and 1´-hydroxymidazolam were obtained from Toronto Research Chemicals (Toronto, ON, Canada). β-NADP^+^, D-glucose 6-phosphate, and D-glucose 6-phosphate dehydrogenase were purchased from Oriental Yeast (Tokyo, Japan). The pooled liver microsomes from humans (mixed gender, Pool of 50), cynomolgus monkeys (six males, sexually mature), marmosets (14 males, sexually mature), rats (Sprague Dawley, 132 males, 8–10 weeks old), and mice (CD-1, 530 males, 11 weeks old) were purchased from Sekisui Xenotech (Lenexa, KS, USA). All commercially prepared reagents used were of the highest quality.

***Establishment of embryonic stem (ES) cells from NOG mice***

All animals used in this study were maintained in the Central Institute for Experimental Animals (CIEA) under specific pathogen-free conditions. All experiments were performed in accordance with institutional guidelines (090014 and 17017A), which were approved by the Animal Experimentation Committee of CIEA. The institutional guidelines are in compliance with the ARRIVE guidelines. Embryonic stem cells (ES) were established from NOG mice according to a standard procedure using a medium containing a two-inhibitor cocktail (hereafter designated as 2i) ^1^. Female NOG mice were placed in cages with male NOG mice (1:1) in order to copulate. After 29.5 h, they were examined for the presence of the vaginal plug. Morula/blastocyst-stage embryos were collected on the morning of day 3 of pregnancy by flushing each uterine horn with M2 medium (Thermo Fisher Scientific). After zona pellucida were removed with acidic Tyrode’s solution, the embryos were cultured on gelatin-coated 4-well plates covered with a feeder layer of mitomycin C-treated mouse embryonic fibroblasts (MEFs) isolated from C57BL/6J Jcl mice (CLEA Japan) in KnockOut D-MEM (Thermo Fisher Scientific) supplemented with 2 mM GlutaMAX (Thermo Fisher Scientific), 1% non-essential amino acid (NEAA; Thermo Fisher Scientific), 0.1 mM 2-mercaptoethanol (2-ME; Sigma-Aldrich, MO, USA), 20% fetal bovine serum (FBS; Hyclone Laboratories, Utah, USA), 1,000 U/mL leukemia inhibitory factor (LIF; ESGRO, Sigma-Aldrich, MO, USA), and 2i consisting of 1 µM PD0325901 (Cayman Chemical Co., MI, USA) and 4 µM CHIR99021 (Cayman Chemical Co.). The inner cell mass outgrowth was picked up by glass capillary, dissociated into clumps of few cells with trypsin, and transferred onto fresh substrates. ES-like colonies were picked up and expanded by subsequent passages to establish NOG-ES cell lines.

***Chromosomal analysis***

The chromosome number of established ES cell lines was checked at passages 6 to 7. NOG-ES cells in the sub-confluent phase were collected after treatment with 0.25% trypsin-EDTA. The cells were washed in phosphate-buffered saline and placed on gelatin-coated plates in defined KnockOut D-MEM medium by adding colcemid (Nacalai Tesque) (final concentration: 0.02 µg/mL) and incubated for 30 min at 37 °C. The cells were centrifuged at 1,200 rpm for 5 min at room temperature, and the supernatant was removed. The pellet was resuspended in 1 mL hypotonic solution (0.56% KCl) and incubated for 20 min at room temperature. Cell suspensions were fixed with 9 mL of freshly prepared Carnoy fixative (methanol: acetic acid 3:1) by adding it drop by drop on ice. The cells were then centrifuged at 1,200 rpm for 5 min. Metaphase spreads were prepared by dropping the cells in a fixative onto glass slides after dispersing the cell depression by tapping. For chromosome counting, the slides were stained with 4% Giemsa solution (Thermo Fisher Scientific), and 25 metaphase cells were analyzed under a microscope for each clone.

***Production of Chimeric Mice and Confirmation of Germline Transmission of NOG-ES Cells***

NOG-ES cells were aggregated with zona-free 8-cell embryos to form chimeras. The recipient 8-cell embryos were recovered from the oviduct of C57BL/6J females on embryonic day 2.5 and incubated with acidified Tyrode’s solution to remove the zona pellucida. NOG-ES cells (20 to 30 cells) were collected and cultured with zona-free 8-cell embryos in modified Whitten’s medium (Thermo Fisher Scientific) to obtain aggregated embryos. Aggregated embryos were then transferred into pseudo-pregnant recipient MCH (ICR) females. Chimeric mice were identified by coat color, and germline transmission was tested by mating male chimeras with female NOG mice.

***Generation of P450 oxidoreductase conditional knockout mice***

All animals used in this study were maintained in the Central Institute for Experimental Animals (CIEA) under specific pathogen-free conditions. All experiments were performed in accordance with institutional guidelines (090014 and 17017A), which were approved by the Animal Experimentation Committee of CIEA. The institutional guidelines are in compliance with the ARRIVE guidelines. Two-step homologous recombination strategies using NOG-ES cells for conditional knockout of the P450 oxidoreductase (*Por*) gene in the liver and small intestine are illustrated in **Supplementary Fig. 1**. The *Por^Flox(Puro)^*-targeting vector was electroporated into the NOG-ES cell line clone NGie4. Puromycin-resistant cells were screened using polymerase chain reaction (PCR) to detect the null allele that deleted the floxed region after transient expression of *Flp* recombinase, and two clones carrying the desired integration in the *Por* locus were identified. Germline transmission was also confirmed (**Supplementary Fig. 2B**). The drug-resistance marker gene was then removed from recombinant ES cells by transient expression of *Cre* recombinase, and NOG-ES clone a5363 with the *Por^Flox^* allele was established. In the second homologous recombination step, the *Flpo* recombinase gene was knocked-in at the first ATG site of the *Cyp3a11* gene to delete floxed *Por* exons 5-15 in the liver and small intestine. During this step (STEP2 in **Supplementary Fig. 1**), the *Cyp3a11^Flpo/CAG-Hyg^*-targeting vector was electroporated into the NOG-ES clone a5363. Hygromycin B-resistant cells were screened using PCR and Southern blotting analysis. Nine clones carrying the desired integration within the *Cyp3a11* locus were identified. A double-recombinant ES cell clone, a5647, was microinjected into C57BL/6J host blastocysts, which were subsequently implanted into pseudo-pregnant recipient MCH (ICR) females to generate chimeric mice (**Supplementary Fig. 2C**). One chimeric male exhibited germline transmission of the *Por^Flox^* and *Cyp3a11^tm1(Flpo)^* alleles (**Supplementary Fig. 2D**). To obtain *Por^Flox^* *Cyp3a11^tm1(Flpo)^* homozygotes, we performed interbreeding. To confirm the successful deletion of the floxed site, the forward 5′- AGGCTATGGTTAGGGCCAGT-3′ (5arm-F) and reverse 5′-TGGAGAAGAGGGGACAAGAA-3′ (3arm-R) primers were used to detect the null allele, which is a 380-bp fragment. Furthermore, the mice were genotyped using PCR with the following primers: forward 5′-TGGTGCCCACATCTATGTCTGC-3′ (5arm-F2) and reverse 5′-CTTAACATAGTCCACAGCCTGG-3′ (3arm-R2) to distinguish between the *Por* wild-type and *Por^Flox^* alleles (**Supplementary Fig. 2E**). This conditional knockout mouse strain was assigned the following genetic designation: NOG-*Por^tm1^Cyp3a11^tm1^*^(^*^Flpo^*^)^*^Jic^* (formally NOD.Cg-*Prkdc^scid^ Il2rg^tm1Sug^* *Por^tm1^ Cyp3a11^tm1^*^(^*^Flpo^*^)^*^Jic^*/Jic). To ensure that the floxed *Por* gene on both alleles was deleted, the floxed *Por* gene on one allele had been deleted in advance. Mice with the *Por* floxed allele and null allele (abridged name: POR cKO mice) were produced by mating female *Por* floxed homozygotes and male *Por* null heterozygotes. The *Por* genotypes were determined using multiplex PCR using the following primers: forward 5′-TTATGTTGAGGCTCTTAGTAACTCG-3′ (mPor-F1) and reverse 5′-AAAGGTGGGTCCAGTCCCTCTTGC-3′ (mPor-R2del) and 5′-TCAGTGACCTTACATGGAAGCTCG-3′ (mPor-R2wild). The sizes of amplicons derived from the wild-type allele (*wt*), floxed allele (*fl*), and null allele (*null*) were 399 bp, 461 bp, and 810 bp, respectively.

***BrdU incorporation assay in the small intestine***

TK-NOG mice were intraperitoneally injected with BrdU (50 mg/kg) for the analysis of epithelial cell proliferation. After 1, 24, 48, or 72 h, the small intestine was harvested and fixed with 4% (v/v) phosphate-buffered formalin (Mildform; Fujifilm Wako Pure Chemical Industries, Ltd., Osaka, Japan) for the preparation of paraffin-embedded tissue sections. BrdU incorporation was visualized by immunohistochemistry using a Leica BOND Max Stainer and the associated Bond Refine Polymer Detection Kit (Leica Microsystems, Tokyo, Japan) with mouse monoclonal anti-bromodeoxyuridine antibody (clone Bu20a, DakoCytomation Denmark A/S, Glostrup, Denmark). The sections were counterstained with hematoxylin. Images were captured using a digital slide scanner (NanoZoomer S60; Hamamatsu Photonics, KK Hamamatsu, Japan).

**Supplementary Information**

***Establishment of NOG-ES cell lines***

ICMs removed from seven blastocysts were plated on a mitomycin C-treated MEF feeder layer and cultured for more than six passages. Six of the seven ICM-derived cells showed flat, packed, and tight colony morphology and a high nucleus-to-cytoplasm ratio, from which an ES cell line (named NOG-ES) was successfully established. The NOG-ES lines had a normal diploid chromosome number (**Supplementary Table 1**). These NOG-ES cell lines exhibited authentic ES cell markers, including Nanog and Oct3/4 (**Supplementary Fig. 2A**). The germline transmission abilities of the two NOG-ES cell lines were confirmed. We aggregated NOG-ES cells with C57BL/6J 8-cell embryos and transferred 160 aggregated embryos into 18 pseudo-pregnant MCH (ICR) mice. Nine chimeric mice (seven males and two females) were obtained with a coat color indicative of the presence of NOG-ES cells (**Supplementary Table 2**). The seven male chimeras were then mated with female NOG mice and produced a total of 68 offspring of white color coat (**Supplementary Table 3**). These results confirmed that NOG-ES cells maintained their capacity for incorporation into the developing embryo and could be transmitted through the germline.

**References**

1 Buehr, M. *et al.* Capture of authentic embryonic stem cells from rat blastocysts. *Cell* **135**, 1287-1298, doi:10.1016/j.cell.2008.12.007 (2008).

**Supplementary Table 1.** Chromosome number in NOG-ES cell lines.

| **Clone** | **Mode of Number** |
| --- | --- |
| NGie1 | 39 (48%) |
| NGie2 | 40 (76%) |
| NGie3 | 40 (76%) |
| NGie4 | 40 (96%) |
| NGie5 | 40 (80%) |
| NGie6 | 40 (88%) |

**Supplementary Table 2.** Generation of chimeric mice.

| **Clone** | **Number of Passages** | **Number of transfers** | **Number of recipients** | **Number of offspring** | **Number of chimeras** |
| --- | --- | --- | --- | --- | --- |
| NGie3 | P7 | 80 | 8 | 7 | 6 |
| NGie4 | P9 | 80 | 10 | 9 | 3 |

**Supplementary Table 3.** Germline transmission of NOG-ES cells.

| **Clone** | **Animal ID** | **Chimeric ratio (%)*** | **Number of offspring** | **Number of germline transmitted offspring** |
| --- | --- | --- | --- | --- |
| NGie3 | NGie3-1 | 100 | 9 | 9 (100%) |
|  | NGie3-2 | 40 | 16 | 16 (100%) |
|  | NGie3-3 | 100 | 16 | 16 (100%) |
|  | NGie3-4 | 30 | 7 | 7 (100%) |
| NGie4 | NGie4-1 | 100 | 9 | 9 (100%) |
|  | NGie4-2 | 80 | 11 | 11 (100%) |
|  | NGie4-3 | 60 | Non | Non |

Non: not obtained offspring, *: Chimeric ratio was estimated by the coat color contribution derived from the NOG-ES cells (albino).

**Supplementary Table 4.** TaqMan probe list for drug metabolism–related gene expression in mice.

| **Species** | **Gene name** | **TaqMan probe ID** |
| --- | --- | --- |
| Mouse | *Cyp1a1* | Mm00487218_m1 |
| Mouse | *Cyp1a2* | Mm00487224_m1 |
| Mouse | *Cyp2a4/Cyp2a5* | Mm00487248_g1 |
| Mouse | *Cyp2a12* | Mm00504878_m1 |
| Mouse | *Cyp2a22* | Mm01614147_m1 |
| Mouse | *Cyp2b9* | Mm00657910_m1 |
| Mouse | *Cyp2b10* | Mm01972453_s1 |
| Mouse | *Cyp2b13* | Mm03052613_s1 |
| Mouse | *Cyp2c29* | Mm00725580_s1 |
| Mouse | *Cyp2c37* | Mm00833845_m1 |
| Mouse | *Cyp2c38* | Mm00658527_m1 |
| Mouse | *Cyp2c39* | Mm04207909_g1 |
| Mouse | *Cyp2c44* | Mm01197188_m1 |
| Mouse | *Cyp2c50* | Mm00663066_gH |
| Mouse | *Cyp2c54* | Mm02602271_mH |
| Mouse | *Cyp2c55* | Mm00472168_m1 |
| Mouse | *Cyp2c70* | Mm00521058_m1 |
| Mouse | *Cyp2d9* | Mm00651731_m1 |
| Mouse | *Cyp2d10* | Mm00731648_m1 |
| Mouse | *Cyp2d13* | Mm01731258_g1 |
| Mouse | *Cyp2d22* | Mm00530542_m1 |
| Mouse | *Cyp2d26* | Mm00472520_m1 |
| Mouse | *Cyp2d40* | Mm01303815_m1 |
| Mouse | *Cyp2e1* | Mm00491127_m1 |
| Mouse | *Cyp3a11* | Mm00731567_m1 |
| Mouse | *Cyp3a13* | Mm00484110_m1 |
| Mouse | *Cyp3a25* | Mm01209536_m1 |
| Mouse | *Cyp3a41a* | Mm00776855_mH |
| Mouse | *Cyp3a44* | Mm01703321_mH |
| Mouse | *Cyp3a59* | Mm01607174_mH |

**Supplementary Table 5.** TaqMan probe list for drug metabolism–related gene expression in humans.

| **Species** | **Gene name** | **TaqMan probe ID** |
| --- | --- | --- |
| Human | *CYP1A2* | Hs00167927_m1 |
| Human | *CYP2B6* | Hs03044634_m1 |
| Human | *CYP2C8* | Hs00258314_m1 |
| Human | *CYP2C9* | Hs00426397_m1 |
| Human | *CYP2C19* | Hs00426380_m1 |
| Human | *CYP2D6* | Hs02576167_m1 |
| Human | *CYP2E1* | Hs00559368_m1 |
| Human | *CYP3A4* | Hs00430021_m1 |
| Human | *CYP3A5* | Hs00241417_m1 |
| Human | *UGT1A1* | Hs02511055_s1 |
| Human | *UGT1A4* | Hs01655285_s1 |
| Human | *UGT1A6* | Hs01592477_m1 |
| Human | *UGT1A9* | Hs02516855_sH |
| Human | *UGT2B4* | Hs02383831_s1 |
| Human | *UGT2B7* | Hs00426592_m1 |
| Human | *UGT2B15* | Hs00870076_s1 |
| Human | *ABCB1* | Hs00184500_m1 |
| Human | *ABCB11* | Hs00184824_m1 |
| Human | *ABCC2* | Hs00166123_m1 |
| Human | *ABCG2* | Hs01053790_m1 |
| Human | *SLC22A1* | Hs00427552_m1 |
| Human | SLC22A7 | Hs00198527_m1 |
| Human | SLC22A9 | Hs00971064_m1 |
| Human | *SLCO1B1* | Hs00272374_m1 |
| Human | *SLCO1B3* | Hs00251986_m1 |

**Supplementary Table 6.** Cumulative urinary excretion of *S*-warfarin and its metabolites for 72 h after intravenous administration (4.0 mg/kg) in wild-type, POR cKO, normal Hu-liver, and POR cKO Hu-liver mice.

| **Mouse** | ***S*-Warfarin (µg)** | **4′-OH (µg)** | **6-OH (µg)** | **7-OH (µg)** | **8-OH (µg)** |
| --- | --- | --- | --- | --- | --- |
| Wildtype mouse A | 2.6 | 7.4 | 2.3 | 4.1 | 0.31 |
| Wildtype mouse B | 3.6 | 7.4 | 2.4 | 5.5 | 0.76 |
| POR cKO mouse A | 15 | 0.52 | 0.17 | 0.37 | 0.011 |
| POR cKO mouse B | 16 | 0.45 | 0.10 | 0.25 | 0.0090 |
| Normal Hu-liver mouse A | 2.6 | 5.1 | 2.4 | 7.7 | 0.27 |
| Normal Hu-liver mouse B | 2.4 | 4.1 | 3.3 | 9.4 | 0.20 |
| POR cKO Hu-liver mouse A | 2.5 | 0.46 | 4.2 | 11 | 0.069 |
| POR cKO Hu-liver mouse B | 1.5 | 0.24 | 2.3 | 16 | 0.20 |

Data were obtained from two individual animals of the wild-type, POR cKO, humanized liver TK-NOG (normal Hu-liver), and POR cKO humanized liver (POR cKO Hu-liver) mice groups. 10-Hydroxywarfarin was not detected in the urine samples of any animal.

**Figure Legends**

**
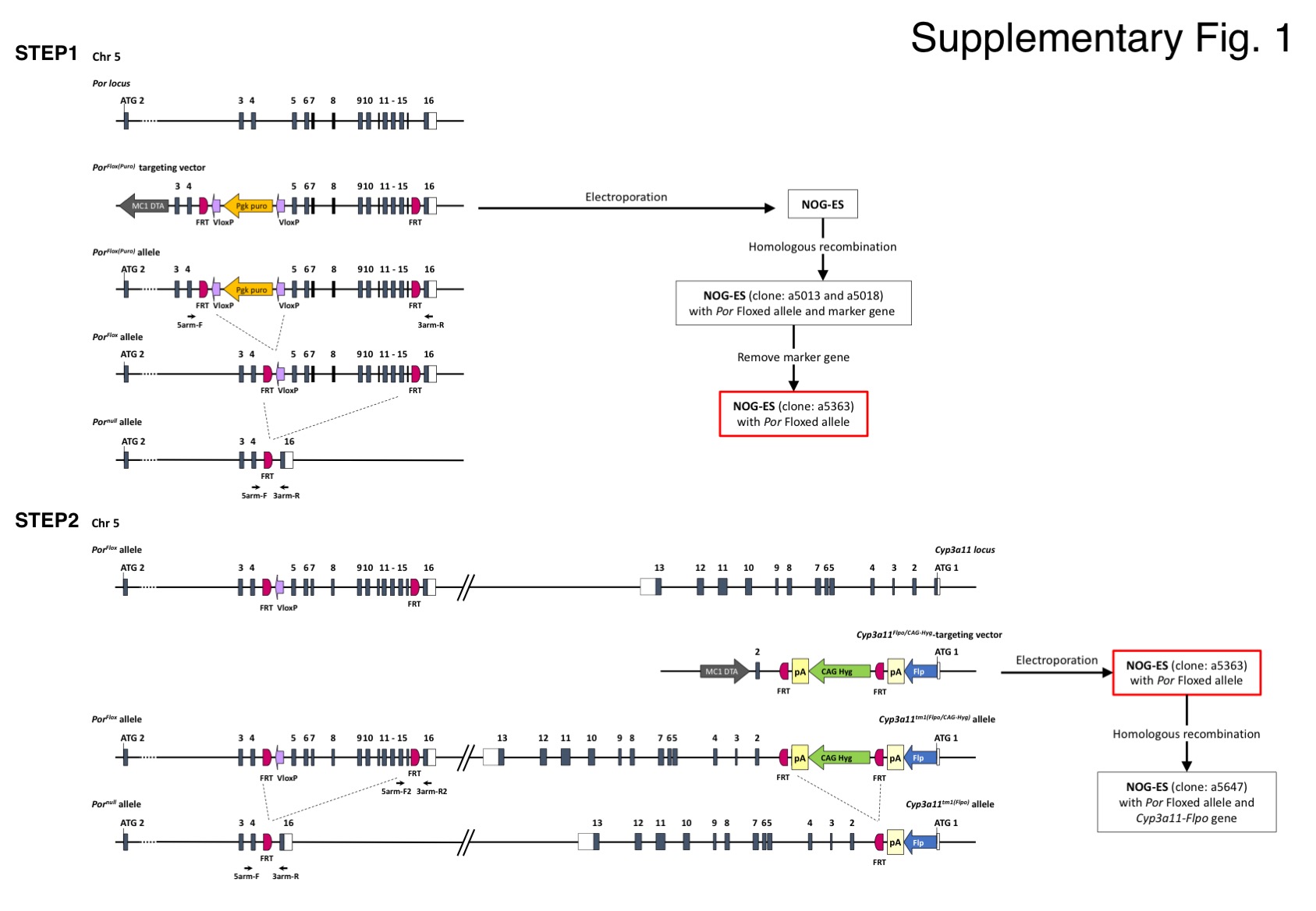
**

**Supplementary Figure 1.** Strategy for P450 oxidoreductase (*Por*) gene conditional knockout. STEP1: Two-step targeting strategy for the generation of POR conditional knockout mice. The *Por^Flox(Puro)^* allele and the derivation of both *Por^Flox^* and *Por^null^* alleles after removal of the PGK-puro cassette through *Cre*-mediated recombination and the removal of the 5th to 15th exons of the *Por* gene after *Flp*-mediated somatic recombination, respectively. STEP2: The *Cyp3a11^tm1(Flpo/CAG-Hyg)^* allele and the derivation of both *Cyp3a11^tm1(Flpo)^* alleles after the removal of the CAG-Hyg cassette following *Flp*-mediated somatic recombination.


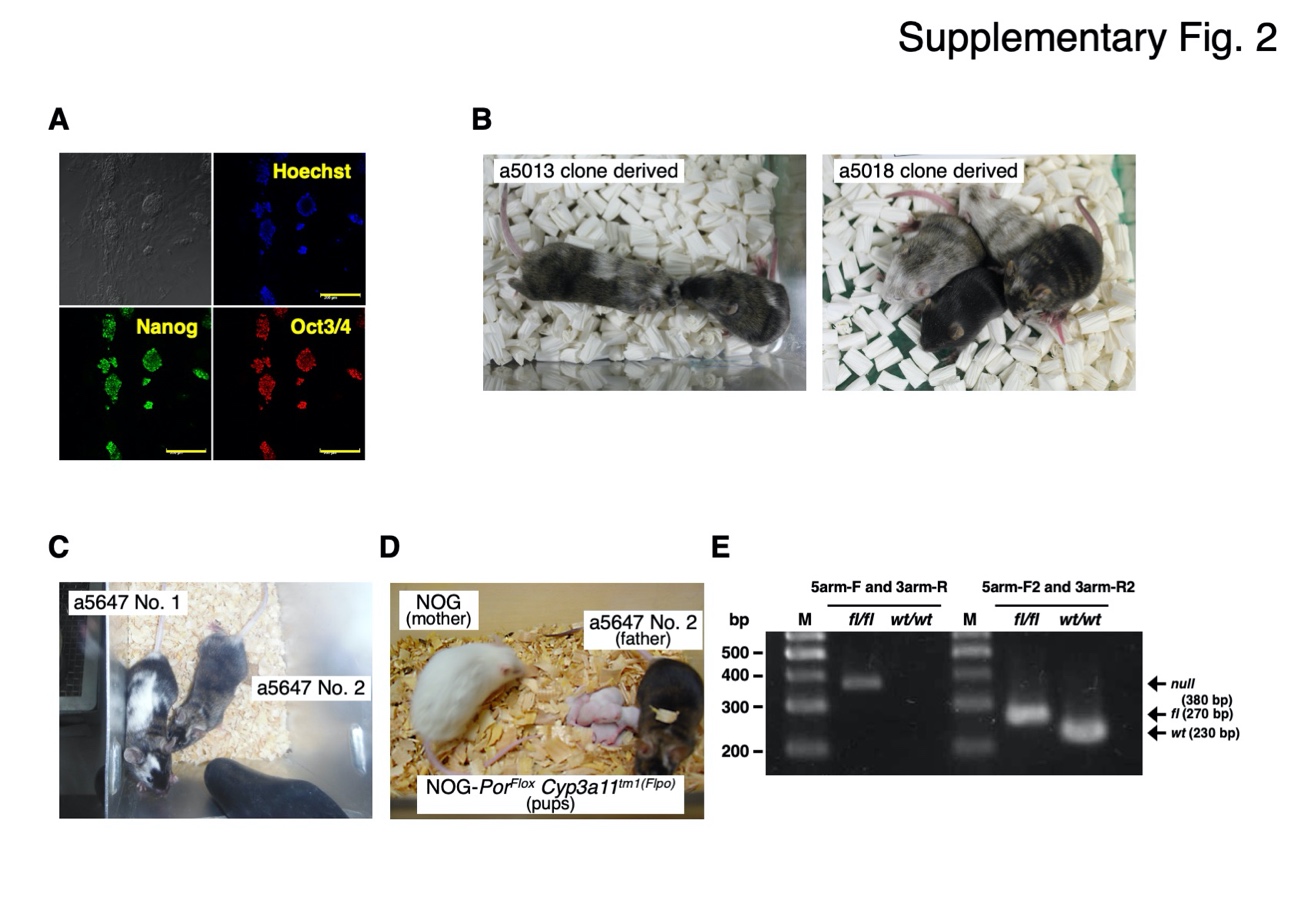


**Supplementary Figure 2.** Generation of POR conditional knockout mice.

(A) Expression of pluripotency markers (Nanog, Oct3/4) in NOG-ES cells (scale bar: 200 µm). (B) Production of chimeric mice with *Por* floxed NOG-ES cells. Chimeric mice generated from the recombinant NOG-ES cells clone a5013 (*left panel*) and a5018 (*right panel*). (C) Production of chimeric mice with *Flp* knocked-in *Por* floxed NOG-ES cells. (D) Confirmation of germline transmission. The double-recombinant NOG-ES cell clone a5647 No. 2 contributed to all the tissues of chimeric mice, including germline. (E) Polymerase chain reaction genotyping of mice using liver biopsy allowing to detect null allele (*null*), *Por* floxed allele (*fl*) and wild-type allele (*wt*). Full-length gel image was presented in Supplemental Fig. 8.

**
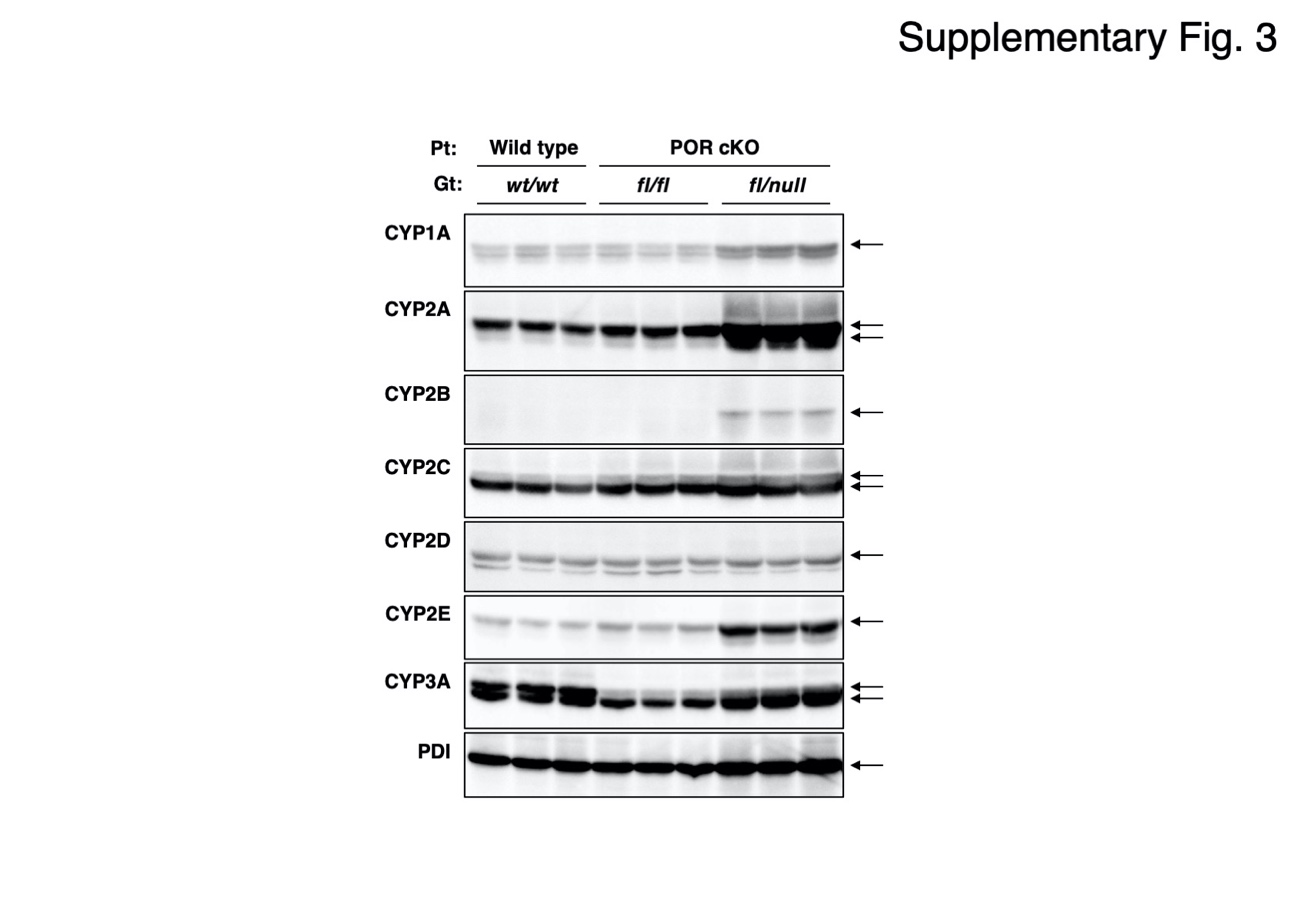
**

**Supplementary Figure 3.** Western blotting analysis of P450 in liver microsomes from wild-type, POR cKO *fl/fl*, and POR cKO *fl/null* mice.

Individual liver microsomes (20 μg/lane, n = 3 for each group) from wild-type, POR cKO *fl/fl*, and POR cKO *fl/null* mice were separated by electrophoresis on a 10% sodium dodecyl sulfate–polyacrylamide gel. Protein expression of mouse P450s (arrows) was determined by immunoblotting with polyclonal antisera against various P450s. Protein disulfide isomerase was used as the loading control. Full-length blot images were presented in Supplemental Fig. 9.

**
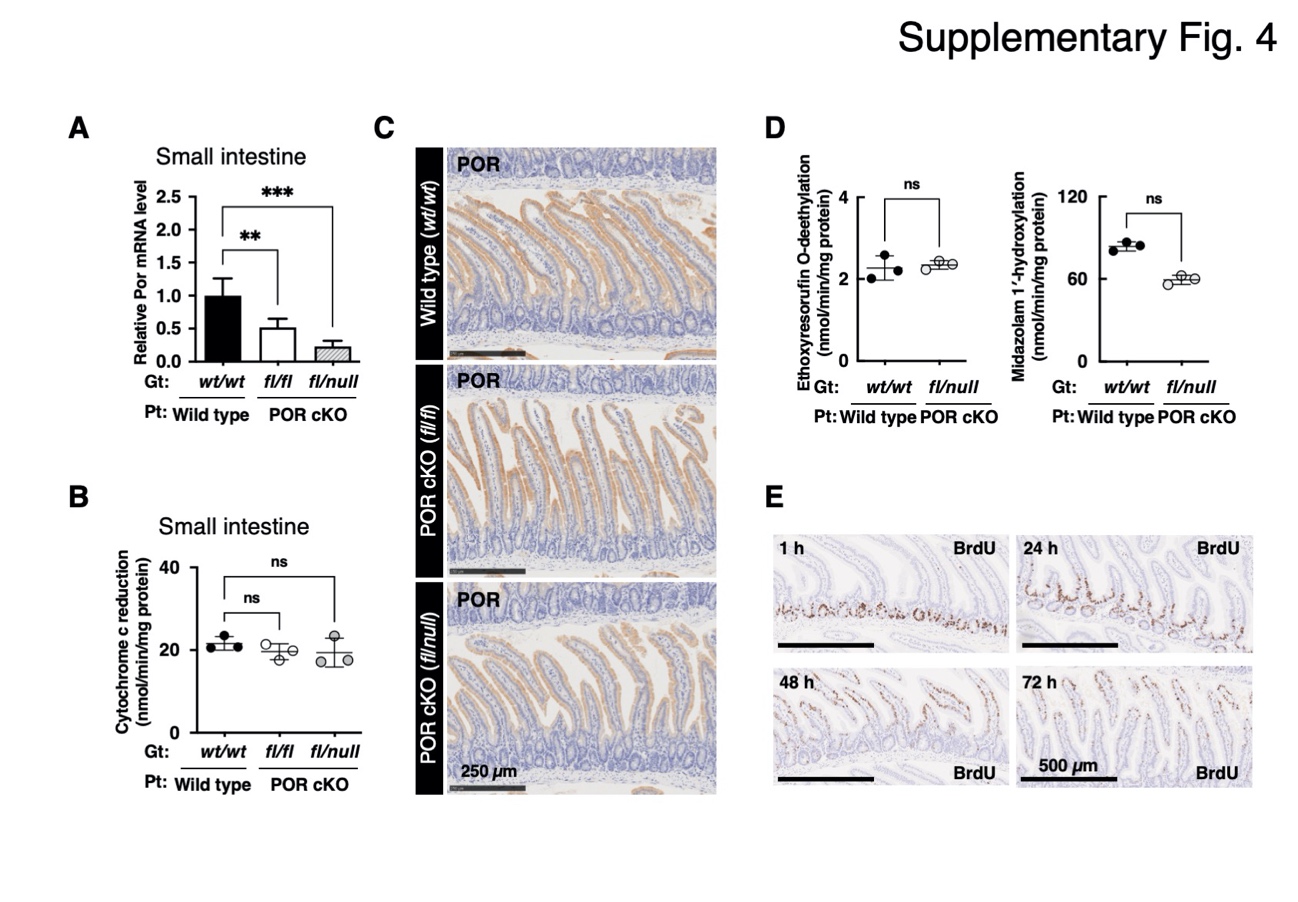
**

**Supplementary Figure 4.** Analysis of the small intestine of POR cKO humanized liver mice.

(A) mRNA expression levels of mouse P450 oxidoreductase genes in the small intestine from wild-type (n = 3), POR cKO *fl/fl* (n = 3), and POR cKO *fl/null* (n = 3) mice were measured using qRT-PCR. Data are presented as the mean ± standard deviation. (B) NADPH-cytochrome c reduction activity in small intestine microsomes from wild-type and POR cKO mice genotype *fl/fl* and *fl/null*. (C) Immunohistochemical staining of Por proteins in the intestine of wild-type, POR cKO *fl/fl*, and POR cKO *fl/null* mice. Scale bar, 250 μm. (D) Ethoxyresorufin (2 μM) and midazolam (100 μM) were incubated with liver microsomes from wild-type and POR cKO mice (0.50 mg/mL) at 37 °C for 10 min. (E) BrdU staining to detect proliferating epithelial cells. BrdU-positive cells in the mouse small intestine were detected by immunohistochemical staining. Mice were injected with BrdU at 50 mg/kg body weight intraperitoneally at 1, 24, 48, and 72 h before sacrifice. The majority of the cells had been shed after 72 h since BrdU administration.

**
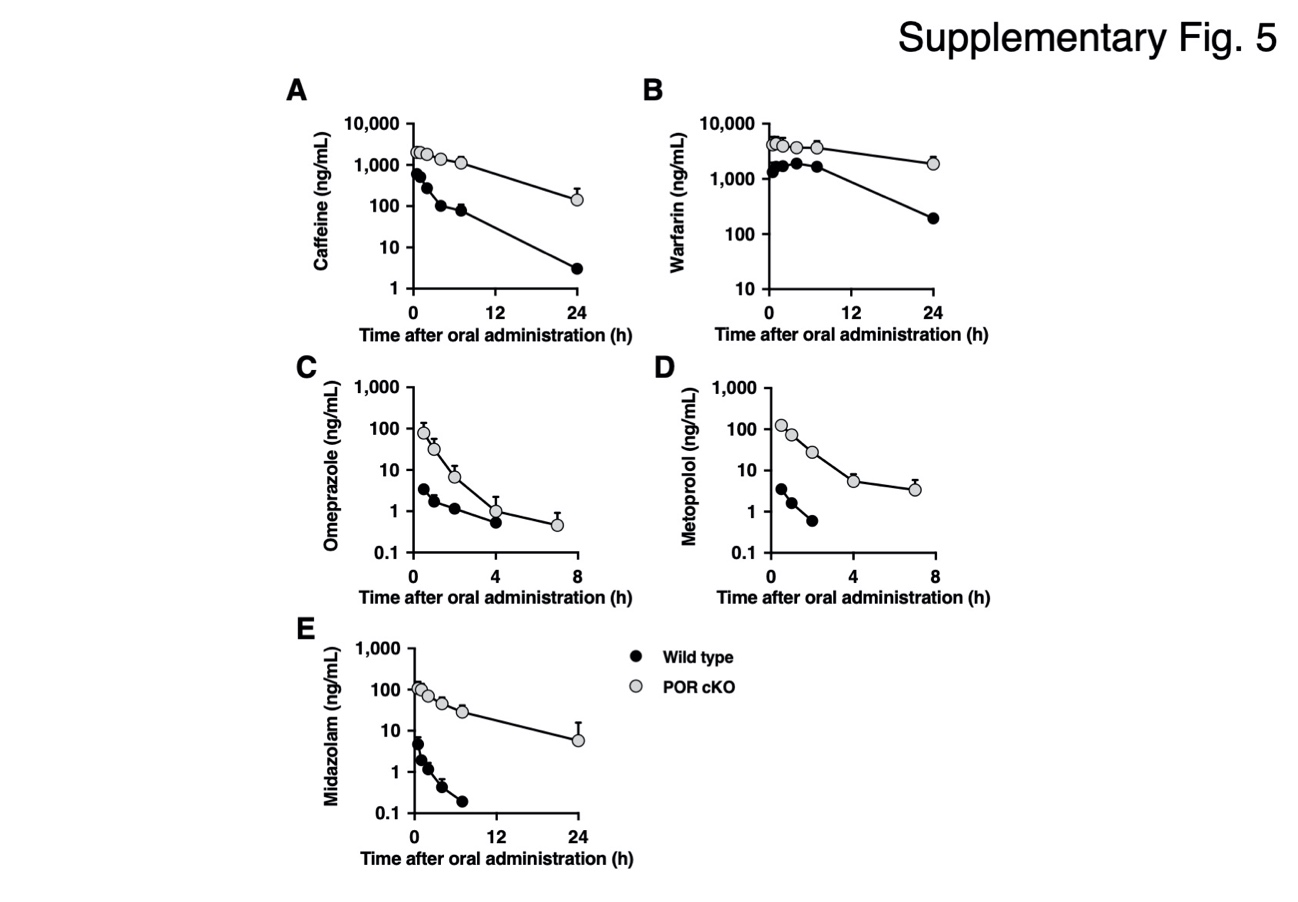
**

**Supplementary Figure 5.** Plasma concentrations of caffeine (A), warfarin (B), omeprazole (C), metoprolol (D), and midazolam (E) in wild-type and POR cKO mice after single simultaneous administrations intravenously (1.0 mg/kg each). Plots and bars are presented as the mean ± SD values of six wild-type and nine POR cKO mice.


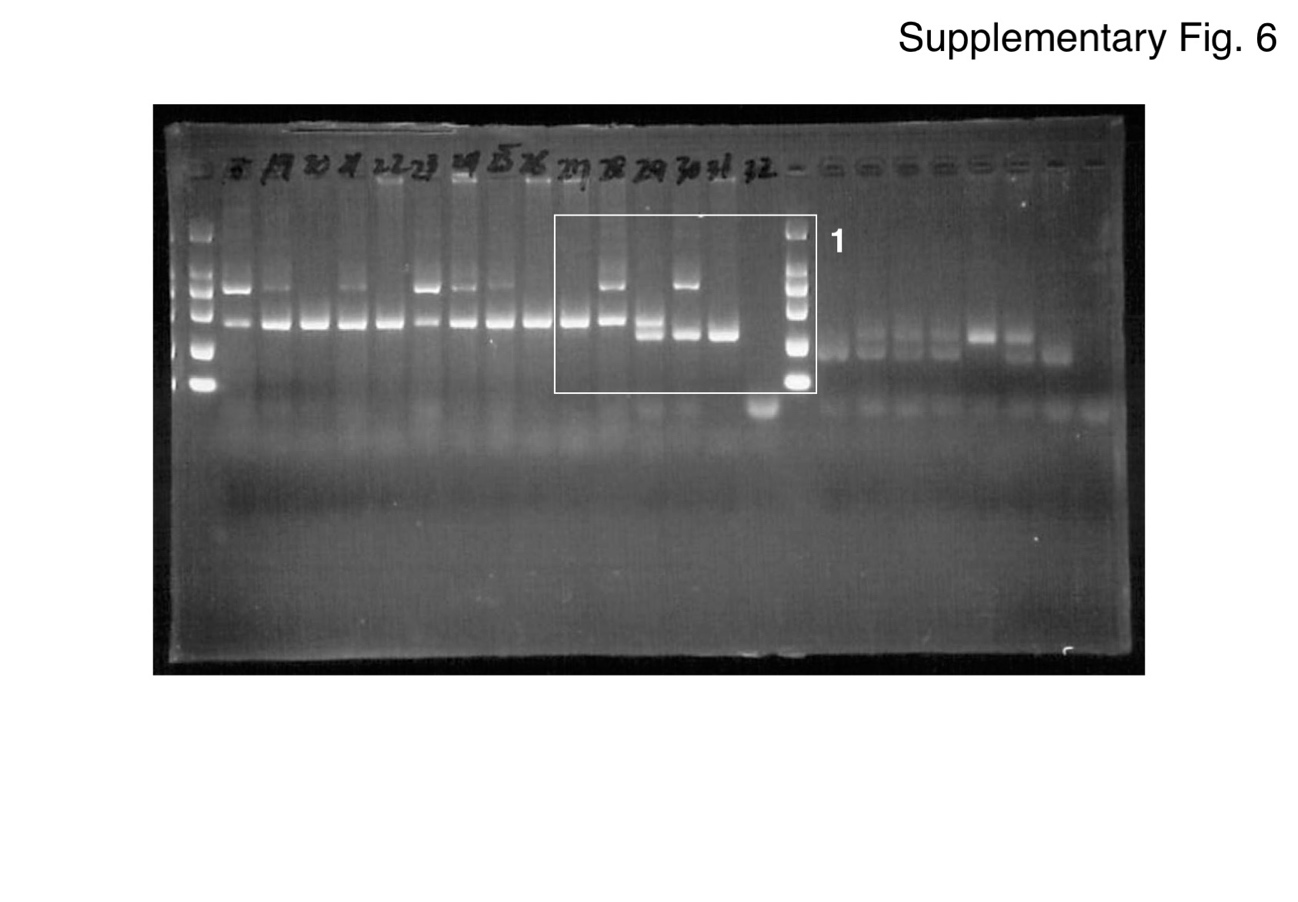


**Supplementary Figure 6.** Full-length gel image before cropping for Figure 2A. Representative PCR genotyping results for POR conditional knockout mice were shown (1).


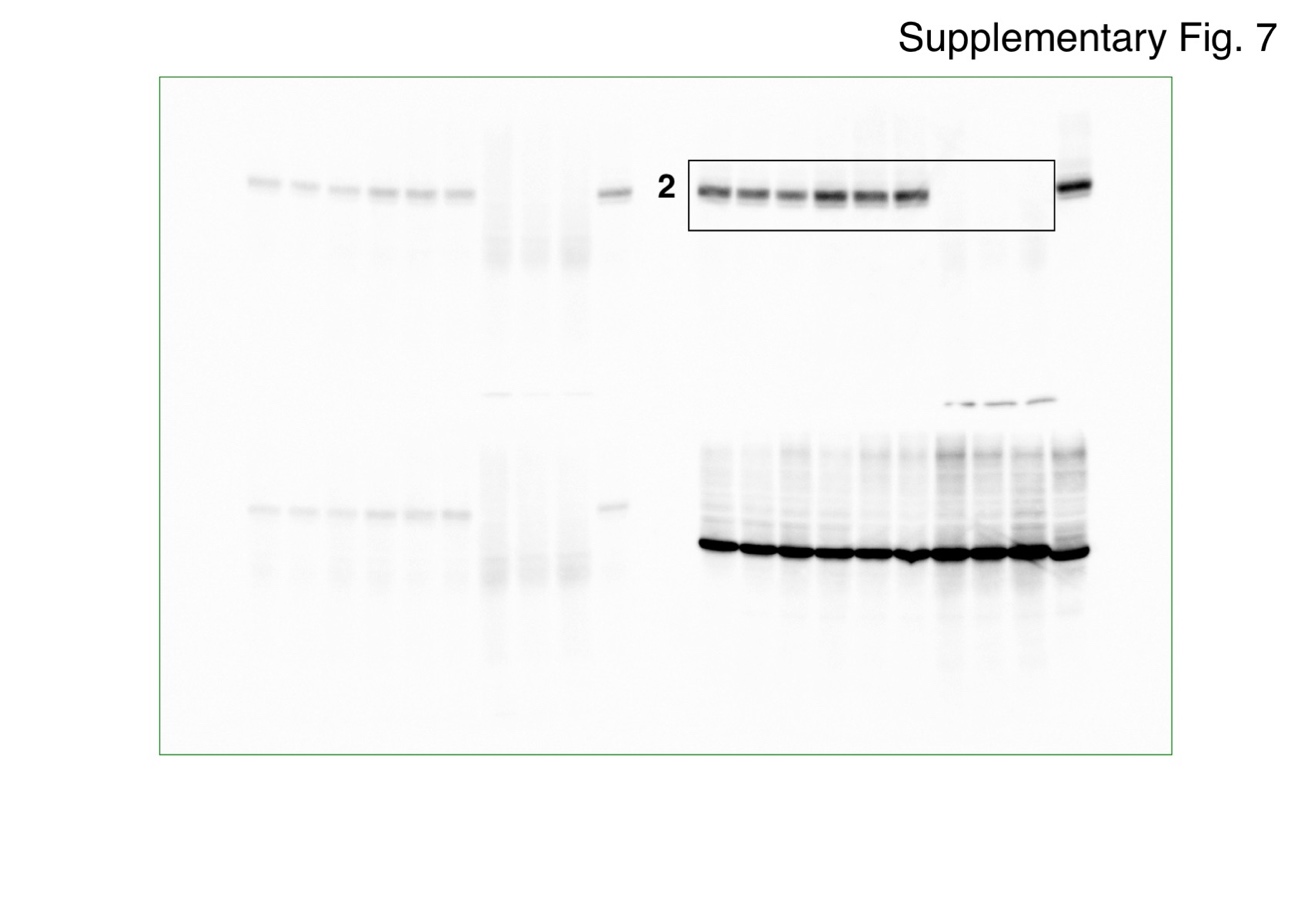


**Supplementary Figure 7.** Full-length blot image before cropping for Figure 2B. Western blot analysis of POR protein in liver microsomes from wildtype, POR cKO *fl*/*fl*, and POR cKO *fl*/*null* mice were shown (2).


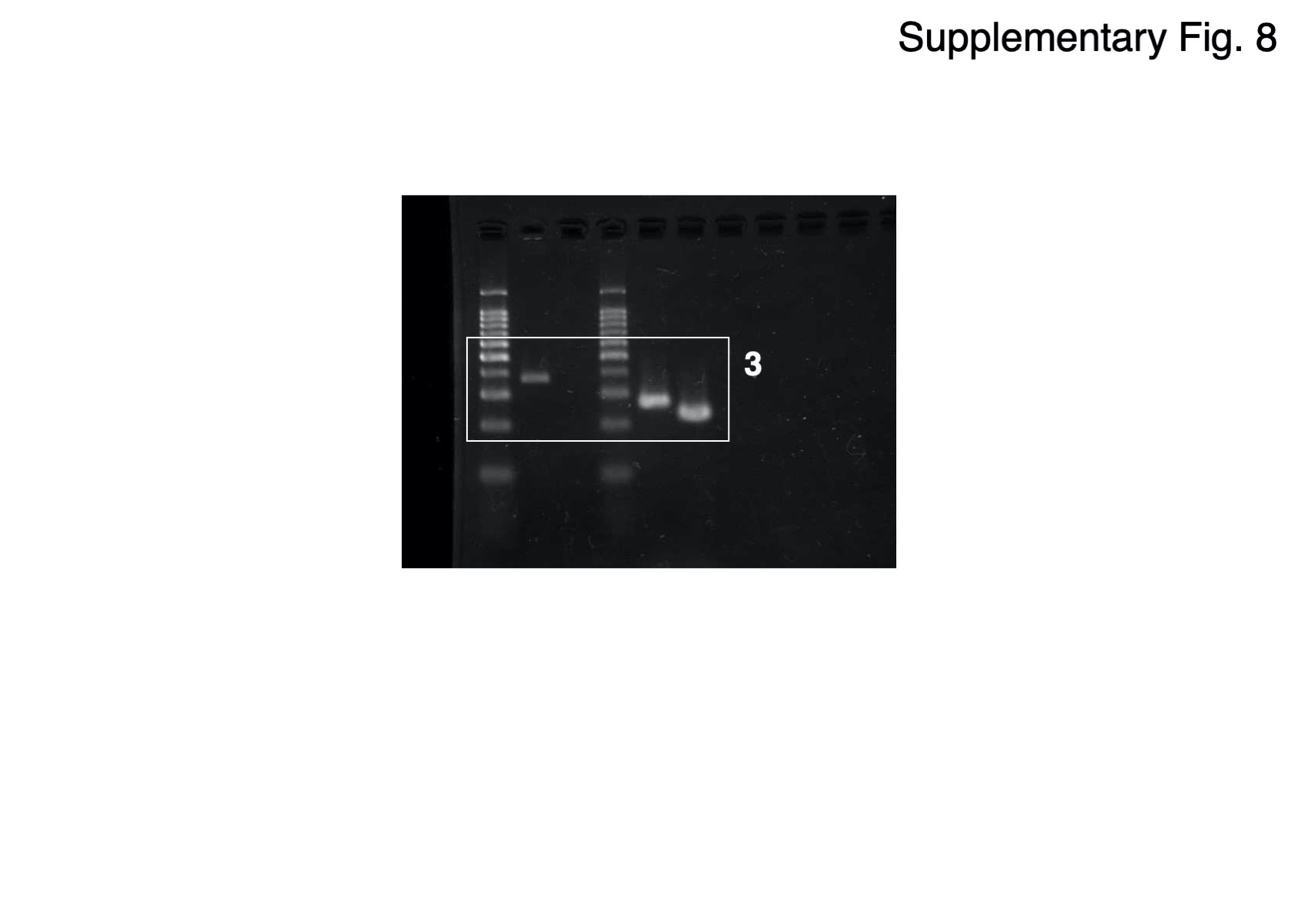


**Supplementary Figure 8.** Full-length gel image before cropping for Supplementary Figure 2E. Polymerase chain reaction genotyping of mice using tail biopsy allowing to discriminate between wild type (*wt*/*wt*) and *Por^Flox/Flox^* (*fl*/*fl*) (3).

**
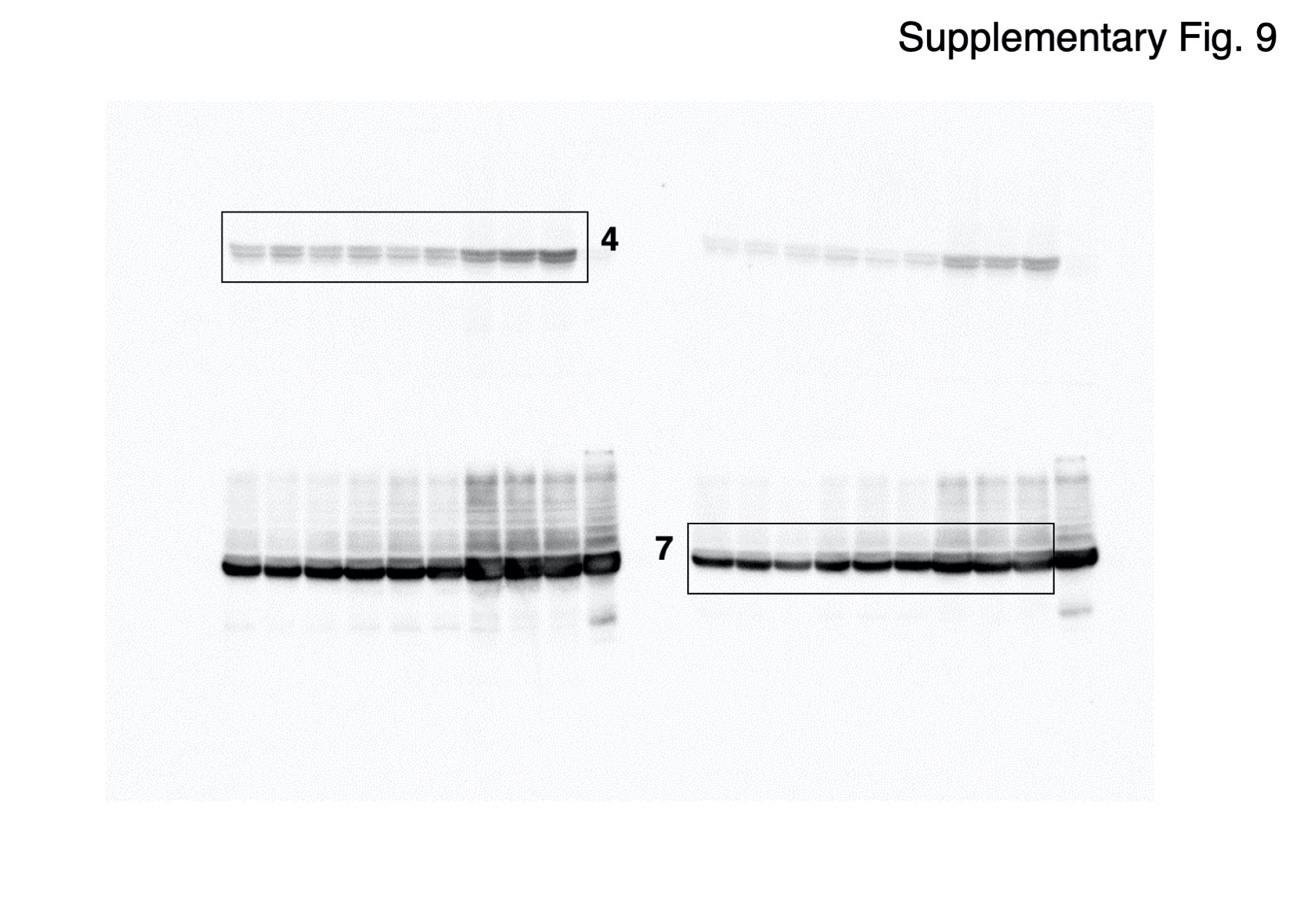

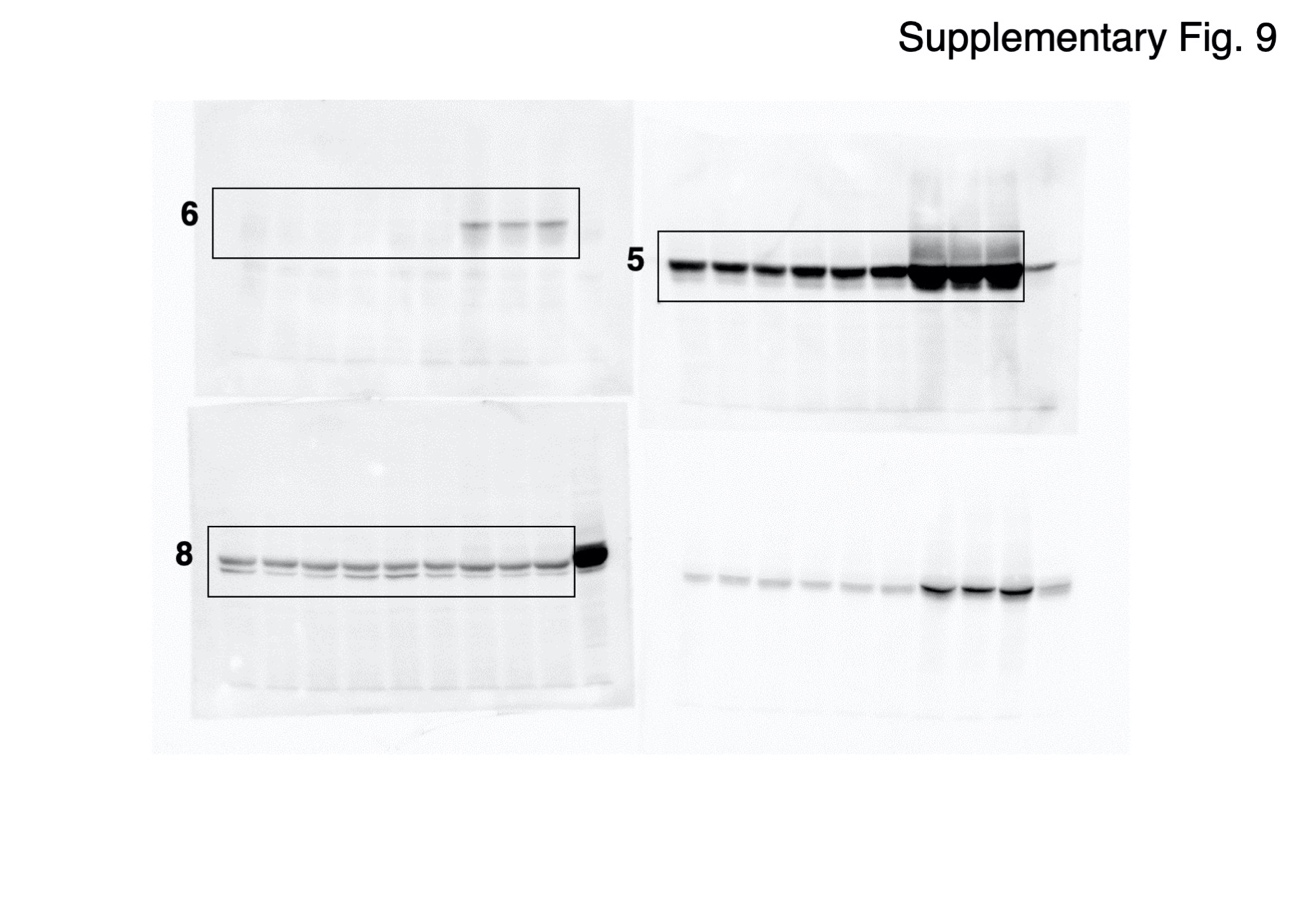

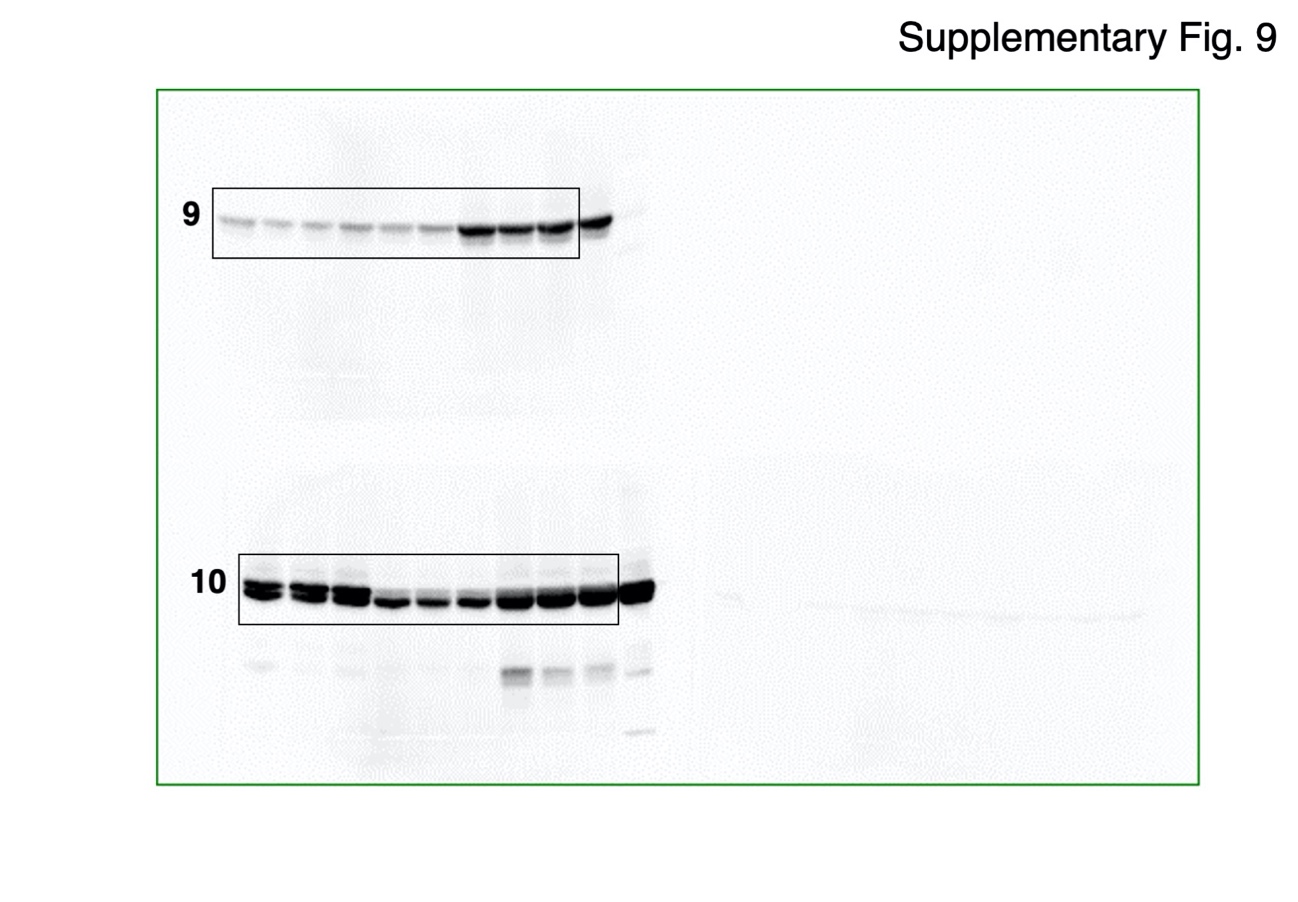

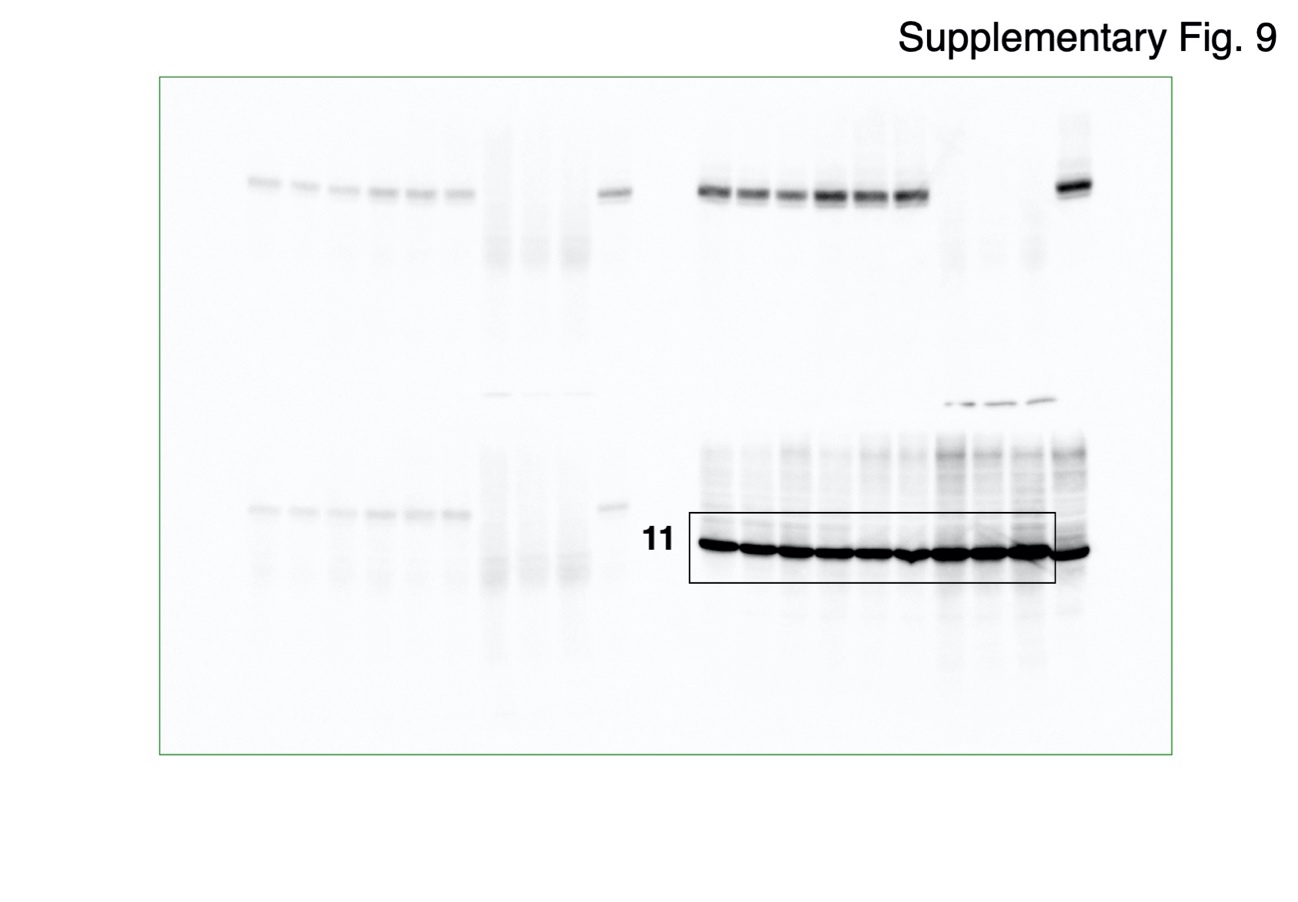
**

**Supplementary Figure 9.** Full-length blot images before cropping for Supplementary Figure 3. Western blot analysis of P450 in liver microsomes from wildtype, POR cKO *fl*/*fl*, and POR cKO *fl*/*null* mice were shown. CYP1A (4), CYP2A(5), CYP2B (6), CYP2C (7), CYP2D (8), CYP2E (9), CYP3A (10) and Protein disulfide isomerase (PDI) (11).
